# Glutamine-derived aspartate is required for eIF5A hypusination-mediated translation of HIF-1α to induce the polarization of tumor-associated macrophages

**DOI:** 10.1038/s12276-024-01214-1

**Published:** 2024-05-01

**Authors:** Dong-Ho Kim, Yoo Na Kang, Jonghwa Jin, Mihyang Park, Daehoon Kim, Ghilsuk Yoon, Jae Won Yun, Jaebon Lee, Soo Young Park, Yu Rim Lee, Jun-Kyu Byun, Yeon-Kyung Choi, Keun-Gyu Park

**Affiliations:** 1 Department of Biomedical Science, Kyungpook National University, Daegu, 41566 South Korea; 2 Department of Forensic Medicine, School of Medicine, Kyungpook National University, Kyungpook National University Hospital, Daegu, 41944 South Korea; 3 Department of Internal Medicine, School of Medicine, Kyungpook National University, Kyungpook National University Hospital, Daegu, 41944 South Korea; 4 Department of Pathology, School of Medicine, Kyungpook National University, Kyungpook National University Chilgok Hospital, Daegu, Daegu, 41404 South Korea; 5 Veterans Medical Research Institute, Veterans Health Service Medical Center, Seoul, 05368 South Korea; 6 Department of Internal Medicine, School of Medicine, Kyungpook National University, Kyungpook National University Chilgok Hospital, Daegu, 41404 South Korea; 7 BK21 FOUR Community‑Based Intelligent Novel Drug Discovery Education Unit, Research Institute of Pharmaceutical Sciences, College of Pharmacy, Kyungpook National University, Daegu, 41566 South Korea; 8 Research Institute of Aging and Metabolism, Kyungpook National University, Daegu, 41566 South Korea

**Keywords:** Cancer metabolism, Cell biology, Cancer microenvironment

## Abstract

Tumor-associated macrophages (TAMs) are vital contributors to the growth, metastasis, and therapeutic resistance of various cancers, including hepatocellular carcinoma (HCC). However, the exact phenotype of TAMs and the mechanisms underlying their modulation for therapeutic purposes have not been determined. Here, we present compelling evidence that glutamine-derived aspartate in TAMs stimulates spermidine production through the polyamine synthesis pathway, thereby increasing the translation efficiency of HIF-1α via eIF5A hypusination. Consequently, augmented translation of HIF-1α drives TAMs to undergo an increase glycolysis and acquire a metabolic phenotype distinct from that of M2 macrophages. Finally, eIF5A levels in tumor stromal lesions were greater than those in nontumor stromal lesions. Additionally, a higher degree of tumor stromal eIF5A hypusination was significantly associated with a more advanced tumor stage. Taken together, these data highlight the potential of inhibiting hypusinated eIF5A by targeting glutamine metabolism in TAMs, thereby opening a promising avenue for the development of novel therapeutic approaches for HCC.

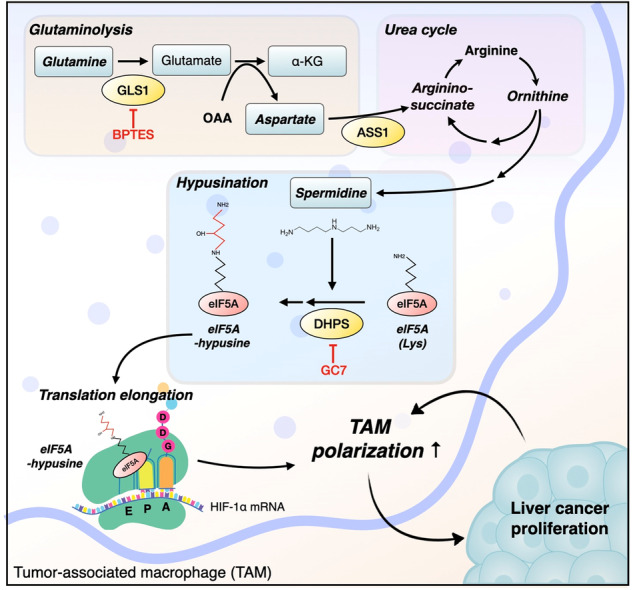

## Glutamine metabolism in TAMs: targeting HIF-1α translation for HCC

This research examines how a substance derived from glutamine (an amino acid) called aspartate influences tumor-associated macrophages (TAMs - immune cells found in many cancers). Led by Dr. Keun-Gyu Park, the team discovered that aspartate from glutamine encourages TAMs to support the growth of hepatocellular carcinoma (HCC - a liver cancer). This process is aided by a protein, eIF5A. The team performed tests on mice and human cells and studied tissue samples from HCC patients. They discovered that blocking eIF5A’s function in TAMs could halt tumor growth. The research suggests that focusing on this metabolic process in TAMs could be a potential treatment strategy for HCC. The results also hint at possible future applications for treating other cancers.

This summary was initially drafted using artificial intelligence, then revised and fact-checked by the author.

## Introduction

Tumor-associated macrophages (TAMs) are immune cells that account for a large proportion of the cells in the tumor microenvironment (TME) in many cancers, including hepatocellular carcinoma (HCC)^1^. High numbers of TAMs in the HCC microenvironment are indicative of poor clinical outcomes^2–4^; therefore, inhibiting TAM polarization may be an effective therapeutic strategy for HCC. TAMs can induce the proliferation, angiogenesis, and migration of HCC cells by secreting growth factors or cytokines such as platelet-derived growth factor (PDGF), matrix metalloproteinases, vascular endothelial growth factor (VEGF), interleukin (IL)-6, IL-10, and chemokine (C-C motif) ligand 2 (CCL2)^1,5^. TAMs are considered to be M2-like macrophages, which mainly perform anti-inflammatory functions to promote tumor development^6,7^; however, the phenotype of TAMs is generally much more complex than that of M2 macrophages, and the former are regulated by many substances within the TME^8^. This complexity is the reason that the pathways involved in TAM polarization have not been fully elucidated.

Glutamine is enriched in the TME, where it acts as a carbon source to fuel the TCA cycle; it also functions as a nitrogen donor, orchestrating the biosynthesis of alanine, aspartate, and serine^9–11^. Accumulating evidence suggests that glutamine metabolites play a role in both the M1 and M2 polarization of macrophages^12–15^. Glutamine metabolism in M1 macrophages promotes the accumulation of succinate by replenishing TCA cycle intermediates for anaplerosis, further improving the stability of HIF-1α, which regulates the polarization of M1 macrophages^12^. Glutamine metabolism in M2 macrophages is essential for supporting TCA cycle activity and providing substrates for N-glycosylation, thereby enabling the glycosylation of M2-related proteins and promoting polarization to the M2 phenotype^13,14^. The synthesis of glutamate from glutamine via glutaminase (GLS) and oxaloacetate is catalyzed by glutamic-oxaloacetic transaminase 1 (GOT1) to produce α-ketoglutarate (α-KG) and aspartate^16^. The production of glutamine-derived α-KG also results in macrophage polarization to the M2 phenotype by regulating epigenetic reprogramming of M2 genes^17^. In contrast, glutamine-derived aspartate is used as a precursor for protein and nucleotide synthesis and for redox homeostasis, which fuels tumorigenesis^18^; however, the role of glutamine-derived aspartate in TAM polarization remains to be defined. Given that HIF-1α in TAMs is upregulated upon exposure to the TME^19,20^, it is necessary to evaluate the impact of glutamine-derived aspartate on the HIF-1α level in TAMs, as well as the mechanism by which aspartate metabolism engenders the tumor-promoting capacity of TAMs.

The urea cycle is coupled to the TCA cycle through the aspartate-argininosuccinate shunt; thus, aspartate can be incorporated into the urea cycle via argininosuccinate synthetase 1 (ASS1), which serves as a key enzyme for arginine production^21,22^. Arginase catalyzes the conversion of arginine to ornithine, which serves as an intermediate during the synthesis of urea and polyamines^23^. Polyamines such as putrescine, spermidine, and spermine, all of which are organic molecules containing two or more amino groups, are found in all eukaryotic cells^23^. Intracellular polyamines play a variety of roles in cell proliferation, gene expression, autophagy, necrosis, and apoptosis^24^. In addition, polyamines regulate protein elongation through the hypusination of eukaryotic initiation factor 5 A (eIF5A), which is mediated by deoxyhypusine synthase (DHPS). Previous studies have reported conflicting results with regard to whether blocking eIF5A hypusination suppresses the activation of M1 or M2 macrophages^25,26^; thus, the exact role of eIF5A hypusination in macrophages remains to be elucidated.

Here, we show that glutamine-derived aspartate in TAMs increases the production of spermidine through the polyamine synthesis pathway, thereby increasing the translation efficiency of HIF-1α through eIF5A hypusination and endowing TAMs with a metabolic phenotype distinct from that of M2 macrophages. Our findings suggest that inhibiting hypusinated eIF5A by targeting glutamine metabolism in TAMs may be a promising therapeutic strategy for HCC.

## Materials and methods

### Patients and specimens

Samples of HCC and adjacent nontumor tissues were obtained from 205 patients who underwent surgical resection at Kyungpook National University Hospital in Daegu, Korea, between 2005 and 2010. HCC was diagnosed and treated according to the American Association for the Study of Liver Diseases guidelines^27^. Patients who had received preoperative anticancer treatments, such as transarterial chemoembolization or local ablation therapy, were excluded. Clinical data (age, sex, tumor size, tumor number, laboratory results, and etiology of underlying liver disease) were obtained by reviewing patients’ medical records. The study was approved by the Institutional Review Board of Kyungpook National University Hospital (KNUH-2014-04-056-001).

### Cell line culture and preparation of conditioned medium (CM) from liver cancer cells

The murine HCC cell line Hepa1-6 (ATCC, VA, USA, #CRL-1830) was cultured in Dulbecco’s modified Eagle’s medium (DMEM; HyClone, Logan, UT, USA, #SH30243) supplemented with 10% fetal bovine serum (FBS; HyClone, #SH30919.03) and 1% penicillin/streptomycin (P/S; Gibco, Grand Island, NY, USA, #15140-122). The human liver cancer cell lines SK-Hep-1 (ATCC, #HTB-52) and Huh-7 (KCLB, Seoul, Korea, #60104), and the HEK293T cell line (ATCC, #CRL-3216) were cultured in DMEM containing 10% FBS and 1% P/S. Other human liver cancer cell lines, i.e., HepG2 (ATCC, #HB-8065) and Hep3B (ATCC, #HB-8064), were cultured in Eagle’s minimum essential medium (EMEM; ATCC, #30-2003) supplemented with 10% FBS and 1% P/S. All the cells were incubated at 37 °C in a 5% CO_2_ atmosphere. To generate conditioned medium (CM) from liver cancer cells, 9 × 10^6^ cells were seeded into a 150 mm dish. The next day, the medium was removed, and the cells were incubated for 24 h with serum-free medium. The CM was collected and passed through a 0.2 μm pore filter.

### Isolation and culture of peritoneal macrophages (PMs)

PMs were isolated from 7–8-week-old male C57BL/6 mice. The mice were injected intraperitoneally with 1 mL of 3% thioglycolate broth (Sigma, St. Louis, MO, USA, #T9032) and then sacrificed 4 days later. PBS (2 injections, 5 mL each) was injected into the peritoneal cavity prior to gentle massage and aspiration of the fluid. Harvested cells were centrifuged for 5 min at 600 × *g* and 4 °C. Next, the cells were cultured in RPMI 1640 medium (HyClone, #SH30027) and allowed to adhere to the dish for 1 h at 37 °C. Nonadherent cells were removed by washing three times with warm PBS. To polarize TAMs, PMs were cultured for 24 h in Hepa1-6-CM with or without BPTES (20 μM; Selleckchem, Houston, TX, USA; #S7753), GC7 (50 μM; Selleckchem, #S2961), dimethyl-2-ketoglutarate (DKG; 1 mM; Sigma, #349631), glutathione reduced ethyl ester (2 mM; Sigma, #G1404), aspartate (20 mM; Sigma, #A7219), and spermidine (20 µM; Sigma, #S2616). For M2 polarization, PMs were treated for 24 h with IL-4 (20 ng/mL; ProSpec-Tany TechnoGene Ltd., Ness Ziona, Israel, #CYT-282). For low-glutamine treatment, PMs were cultured for 24 h in a 1:9 (v/v) mixture of Hepa1-6-CM and glutamine-depleted RPMI 1640 medium (Welgene, Gyeongsan, Korea).

### Animal experiments

C57BL/6 and BALB/c nude mice were purchased from Dooyeol Biotech (Seoul, Korea). Briefly, 5 × 10^6^ Hepa1-6 cells were injected subcutaneously into the bilateral flanks of 7-week-old male C57BL/6 mice. The mice were then randomized into two sets of two groups to undergo two distinct treatment conditions (*n* = 12 in the vehicle control group and *n* = 12 in the BPTES group; *n* = 12 in the vehicle control group and *n* = 12 in the GC7 group). When the tumor volume reached approximately 100 mm^3^, each mouse received the vehicle control, BPTES (12.5 mg/kg) or GC7 (15 mg/kg) daily for 12 or 14 days via intraperitoneal injection. For the allograft model with implantation of Hepa1-6 cells mixed with TAMs, PMs were transfected with scrambled siRNAs, mouse siGLS, or sieIF5A and then polarized into TAMs by culture in Hepa1-6-CM. A mixture of Hepa1-6 cells and TAMs (6:1) was then injected subcutaneously into the bilateral flanks of 4-week-old male BALB/c nude mice (*n* = 10 in the scrambled siRNA, siGLS, and sieIF5A groups). To confirm the expression of TAM markers in the tumors, tumors were harvested from random mice in each group on Day 6 post-inoculation. Tumors were measured every 2 days using calipers, and the tumor volume was calculated as length × width^2^ × 0.5 (mm^3^). All animal procedures were performed in accordance with guidelines approved by the Institutional Animal Care and Use Committee of Kyungpook National University.

### Isolation of monocytes from human peripheral blood mononuclear cells (PBMCs) and differentiation of TAMs

Blood samples obtained from healthy donors were diluted in PBS containing 2% FBS, transferred to a SepMate™ PBMC isolation tube (STEMCELL Technologies, Vancouver, BC, Canada, #86450) containing Lymphoprep™ (STEMCELL Technologies, #07801), and centrifuged at 1200 × *g* for 20 min. The enriched cells were washed two times with PBS containing 2% FBS. Then, monocytes were isolated from the PBMCs using a Pan Monocyte Isolation Kit (Miltenyi Biotec, Bergisch Gladbach, Germany, #130-096-537) according to the manufacturer’s instructions. Briefly, cells were resuspended in MACS buffer, and a mixture of FcR blocking reagent and a biotinylated antibody cocktail was added. Then, monocytes were separated from nonmonocytes using magnetic labeling with biotinylated antibodies and anti-biotin microbeads. For TAM differentiation, monocytes were maintained in a 1:1 (v/v) mixture of CM from liver cancer cells and RPMI 1640 medium supplemented with 10% human serum (Sigma, #H5667), 1% P/S, and 20 ng/ml human macrophage colony-stimulating factor (M-CSF; Sigma, #SRP3110). The medium was changed after 3 days, and the cells were harvested on Day 5.

### Western blot analysis

Proteins were extracted using RIPA buffer (Thermo Fisher Scientific, Waltham, MA, USA, #89900) supplemented with phenylmethylsulfonyl fluoride (PMSF; VWR International, Radnor, PA, USA, #0754), aprotinin protease inhibitor (Sigma, #78432), leupeptin protease inhibitor (Sigma, #78435), and a phosphatase inhibitor (Sigma, #P0044) and incubated on ice for 20 min. Then, the cell lysates were cleared via centrifugation at 17,000 × *g* and 4 °C for 20 min. The total protein concentration was determined using the Pierce BCA assay (Thermo Fisher Scientific, #23227). Proteins in the cell lysates were resolved via 10% SDS‒PAGE and transferred to PVDF membranes (Millipore Corporation, Bedford, MA, USA; #IPVH00010). The membranes were blocked with 5% skim milk prepared in Tris-buffered saline containing 0.1% Tween 20 (TBS-T) for 1 h and then incubated with primary antibodies at 4 °C overnight. After three washes in TBS-T, the membranes were incubated with HRP-conjugated secondary antibodies. The primary and secondary antibodies used are detailed in Supplementary Table 2.

### Quantitative RT‒PCR

Total RNA was extracted from cells using QIAzol lysis reagent (Qiagen, Hilden, Germany, #79306). RNA was quantified using a NanoDrop spectrophotometer (Thermo Fisher Scientific). cDNA was subsequently synthesized using the RevertAid First Strand cDNA Synthesis Kit (Thermo Fisher Scientific, #K1622) and was then amplified in a 7500 Fast Real-Time PCR System (Applied Biosystems, Foster City, CA, USA) with SYBR Green PCR Master Mix (Applied Biosystems, #4368708). The expression of the target mRNAs was normalized to that of 36B4 mRNA. The sequences of the primers used are shown in Supplementary Table 3.

### Intracellular metabolite assays

To assess the intracellular levels of glutamine, aspartate, and ornithine, equal numbers of M0 macrophages, M2 macrophages, and TAMs in the presence/absence of BPTES or aspartate were homogenized, and the glutamine (Biovision, Milpitas, CA, USA, #K556), aspartate (Biovision, #K552), and ornithine (Biovision, #K939) contents were analyzed. The content of spermidine in M0 macrophages and TAMs in the presence/absence of BPTES or aspartate was measured via enzyme-linked immunosorbent assay (MyBioSource, San Diego, CA, USA; #MBS2700698).

### Transfection of siRNA

PMs were transfected for 48 h with scrambled siRNA, mouse siASS1, siHIF-1α, siASNS, siOAT, sieIF5A, siGLS, or siDHPS (Bioneer, Daejeon, Korea) using Lipofectamine 3000 (Thermo Fisher Scientific, #L3000015). The sequences of the siRNAs used are shown in Supplementary Table 4.

### Cell counting and viability assays

To evaluate the effect of TAMs on Hepa1-6 tumor cells, PMs were cultured for 24 h in RPMI 1640 medium or Hepa1-6-CM in the presence/absence of BPTES or GC7. Then, the cells were washed with warm PBS and cultured in fresh RPMI 1640 medium for another 24 h. The supernatant was collected and filtered through a 0.2 μm pore filter to generate macrophage-CM. Hepa1-6 cells were cultured in macrophage-CM for 72 h, stained with trypan blue, and counted using a hemocytometer. The trypan blue exclusion assay was conducted to determine whether BPTES, GC7, aspartate, siASS1, or siHIF-1α affected the viability of TAMs.

### Immunohistochemistry

Tumors were extracted at the experimental endpoint and fixed with 4% PFA (Biosesang, Seongnam-si, Korea, #P2031). After fixation, the tumors were embedded in paraffin and sliced into sections, which were deparaffinized. Antigen retrieval was performed using the IHC-Tek Epitope Retrieval Streamer Set (IHC World, Woodstock, MD, #IW-100). The sections were incubated overnight at 4 °C with primary antibodies. Hematoxylin and eosin (H&E) staining was performed to differentiate nuclei from the extracellular matrix and cytoplasm. For immunofluorescence staining, slides were incubated overnight at 4 °C with primary antibodies. The slides were then washed with 1× PBS and stained for 1 h at room temperature with Alexa Fluor 568-conjugated goat anti-rabbit IgG and Alexa Fluor 488-conjugated goat anti-mouse IgG. Nuclei were then stained with DAPI (Vector Laboratories, Burlingame, CA, USA, #H-1800). Quantification of staining was performed using ImageJ software. The primary and secondary antibodies used are detailed in Supplementary Table 2.

### Labeling of nascent proteins and pull-down assay

PMs were cultured for 24 h in Hepa1-6-CM with or without GC7 (50 μM). The medium was then replaced with methionine-free medium (Sigma, #R7513) supplemented with L-cystine (Sigma, #C7602), L-glutamine (Sigma, #G8540), 10% dialyzed FBS (Gibco, #26400-044), and 1% P/S to deplete intracellular methionine reserves. After 30 min, the cells were treated with L-azidohomoalanine (AHA; Click Chemistry Tools, Scottsdale, AZ, USA, #1066-25) for 4 h to label nascent proteins. Cells were lysed in RIPA buffer (Thermo Fisher Scientific, #89900) containing PMSF (VWR International, #0754), aprotinin protease inhibitor (Sigma, #78432), leupeptin protease inhibitor (Sigma, #78435), and a phosphatase inhibitor (Sigma, #P0044) and then incubated on ice for 20 mins. The cell lysates were then centrifuged at 17,000 × *g* and 4 °C for 20 min, after which the supernatants were collected. Nascent proteins were labeled with the Click-&-Go Protein Reaction Buffer Kit (Click Chemistry Tools, #1262) and Biotin-alkyne (PEG4 carboxamide-Propargyl Biotin) (Click Chemistry Tools, #1266-5). Biotinylated proteins were incubated for 1 h at 4 °C with 50 μL of streptavidin-agarose beads (Thermo Fisher Scientific, #20347). Then, 1 mL of PBS/0.1% SDS was added, the mixture was centrifuged at 2500 × *g* for 2 min, and the supernatant was discarded. This step was repeated four times to remove unbound proteins. Purified proteins were further analyzed by western blotting.

### Transfection of plasmid DNA

pRP-CMV-wild-type-HIF-1α-FLAG and pRP-CMV-mutant-HIF-1α-FLAG were purchased from VectorBuilder. HEK293T cells (4 × 10^5^) were seeded into a 6-well plate and incubated for 24 h. Then, 2 μg of plasmid DNA and TransIT-LT1 transfection reagent (Mirus Bio, Madison, WI, #MIR2300) were mixed with Opti-MEM (Gibco, #31985-070) for 20 min at room temperature and were then added to the cells. After 24 h, the cells were washed twice with warm PBS prior to incubation for another 24 h in fresh medium with or without GC7.

### Measurement of the ECAR and OCR

The ECAR and OCR were measured using an XF-24 Extracellular Flux Analyzer (Seahorse Bioscience, North Billerica, MA, USA). Prior to measurement, 2 × 10^5^ PMs were seeded into a Seahorse XF-24 plate (Seahorse Bioscience, #100777-004). The following day, the cells were treated under the indicated conditions. The sensor cartridge was calibrated the day before measurement of the ECAR and OCR. PMs were washed twice with XF RPMI Base Medium (Seahorse Bioscience, #103576-100) and incubated at 37 °C for 1 h in a non-CO_2_ incubator. Glucose (10 μM; Seahorse Bioscience, #103577-100), oligomycin (1 μM; Sigma, #75351), and 2-deoxy-D-glucose (2-DG; 100 mM; Sigma, #D8375) were added at the indicated times during ECAR measurement. Oligomycin (1 μM; Sigma, #75351), CCCP (5 μM; Sigma, #C2759), and a combination of antimycin A (1 μM; Sigma, #A8674) and rotenone (5 μM; Sigma, #R8875) were added at the indicated times during OCR measurement.

### RNA isolation

Total RNA was isolated using TRIzol reagent (Invitrogen). RNA quality was assessed using the Agilent TapeStation 4000 system (Agilent Technologies, Amstelveen, The Netherlands), and RNA was quantified using an ND-2000 spectrophotometer (Thermo Fisher Scientific).

### Library preparation and sequencing

For the control and test RNAs, library construction was performed using the QuantSeq 3′ mRNA-Seq Library Prep Kit (Lexogen, Inc., Austria). In brief, total RNA was prepared, and its 5′ end was hybridized to an oligo(dT) primer containing an Illumina-compatible sequence. Reverse transcription was subsequently performed. After degradation of the RNA template, second-strand synthesis was initiated by a random primer containing an Illumina-compatible linker sequence at the 5′ end. The double-stranded library was purified using magnetic beads to remove all reaction components. The library was amplified to add the complete adapter sequences required for cluster generation. The finished library was purified from PCR components. High-throughput sequencing was performed as single-end 75 sequencing using the NextSeq 550 platform (Illumina, Inc., USA).

### Data analysis

QuantSeq 3′ mRNA-Seq reads were aligned using Bowtie2^28^. Bowtie2 indices were generated from either the genome assembly sequences or the representative transcript sequences prior to aligning the genome with the transcriptome. The alignment file was used to assemble transcripts, estimate their abundances, and detect differential expression. Differentially expressed genes were identified based on counts from unique and multiple alignments using the coverage tool in Bedtools^29^. The RC (read count) data were processed based on the TMM + CPM normalization method using the EdgeR package in R and Bioconductor^30^. Gene classification was based on searches of the DAVID (http://david.abcc.ncifcrf.gov/) and Medline (http://www.ncbi.nlm.nih.gov/) resources. Data mining and graphical visualization were performed using ExDEGA (Ebiogen, Inc., Korea).

### Gene set enrichment analysis (GSEA)

Gene set enrichment analysis (GSEA) was performed to determine whether there were significant differences in gene sets between M0 or M2 macrophages and TAMs. The focus was on the HALLMARK_HYPOXIA and REACTOME_GLYCOLYSIS gene sets. An enrichment score (ES) was calculated for each gene set to represent the degree to which each gene set was overrepresented at the extremes of the ranked list. To account for differences in gene set sizes, the normalized enrichment score (NES) was calculated. The GSEA computations were performed using the GSEA software available from the Broad Institute (http://www.gsea-msigdb.org/gsea/index.jsp). For enrichment analysis, a *p*-value of <0.05 was considered to indicate statistical significance.

### Gene Ontology analysis

The Database for Annotation, Visualization, and Integrated Discovery (DAVID) resource was used to investigate the biological implications of the differentially expressed genes. Specifically, the functional annotation tool in DAVID was used to identify enriched Gene Ontology (GO) terms within the biological process (BP) category, denoted as GOTERM_BP_DIRECT. This approach facilitated the identification of the top 10 biological processes in M0 and M2 macrophages and in TAMs. A filter was applied to isolate genes exhibiting a ≥1.5-fold change in expression. A *p*-value of <0.05 was considered to indicate statistical significance.

### Statistical analysis

Statistical analysis was performed using GraphPad Prism software. Statistical differences were determined by Student’s *t*-test. All data in the graphs are expressed as the means ± SEMs of at least three independent experiments. Clinical data management and statistical analyses were performed using SPSS software (version 29.0.1.0; SPSS, Inc., Chicago, IL). Associations between protein expression levels were evaluated using Pearson’s χ2 test. *P* < 0.05 was considered to indicate statistical significance.

## Results

### TAMs and M2 macrophages exhibit distinct gene expression profiles and functions

**Fig. 1:**
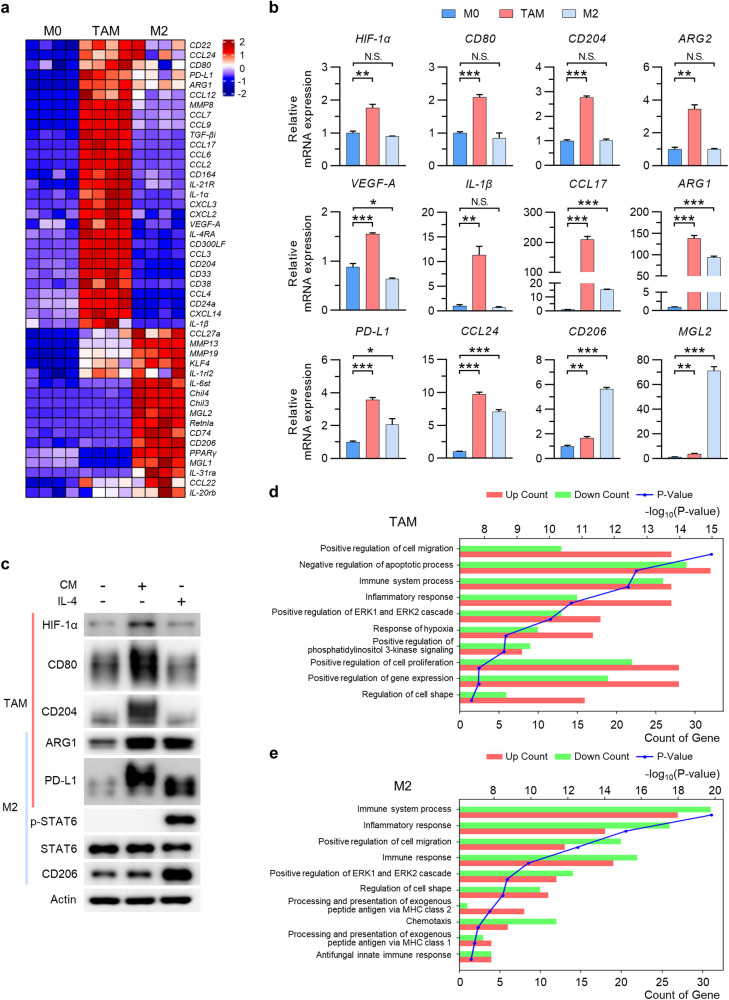
TAMs and M2 macrophages exhibit distinct gene expression profiles. **a** Heatmap of genes expressed by peritoneal macrophages incubated for 24 h in control medium (M0 macrophages), in Hepa1-6 cell conditioned medium (CM) (tumor-associated macrophages; TAMs), or with IL-4 (M2 macrophages). **b** Relative expression of mRNAs encoding the indicated proteins in M0 macrophages, TAMs, and M2 macrophages. **c** Western blot analysis of the indicated proteins in M0 macrophages, TAMs, and M2 macrophages. **d**, **e** Top 10 Gene Ontology biological process (GOBP) terms, ranked according to the significance of changes in gene expression. Comparison of TAMs versus M0 macrophages (**d**) and of M2 macrophages versus M0 macrophages (**e**). Each bar in the graph represents the gene count, where downregulated genes are indicated by green bars and upregulated genes are indicated by red bars. The *p*-value associated with each term is indicated by the blue line. The data are expressed as the mean ± SEM of three independent experiments. N.S. not significant, *p < 0.05; **p < 0.01; and ***p < 0.001. CM conditioned medium.

To assess the differences in the gene expression profiles of TAMs and M2 macrophages, we first induced peritoneal macrophages (PMs) isolated from mice to differentiate into TAMs and M2 macrophages by exposure to conditioned medium (CM) from Hepa1-6 mouse HCC cells and IL-4, respectively. As summarized in the heatmap (Fig. 1a), the expression of several typical M2 markers, including ARG1, KLF4, and CCL24, was increased in TAMs; however, the expression of M1 markers, such as CD38, IL-1α, IL-1β, and CXCL2, was also increased significantly. TAMs also strongly expressed HIF-1α as well as CD80, CD204, PD-L1, VEGF-A, MMP13, and MMP19, indicating the existence of a mixed M1- and M2-like population^31,32^ (Fig. 1a). The expression of typical M2 markers and cytokines, such as Chil3, Chil4, Retnla, MGL1, and MGL2, was increased in M2 macrophages but not in TAMs. Representative genes expressed by TAMs and M2 macrophages (obtained from mRNA sequencing data) were confirmed by qPCR and western blotting (Fig. 1b, c). In addition, we observed that the level of phosphorylated STAT6, which acts downstream of IL-4 receptor signaling, was increased only in M2 macrophages (Fig. 1c). Gene Ontology biological process (GOBP) enrichment analysis revealed that pathways related to cell migration, proliferation, inflammatory responses, and responses to hypoxia were upregulated in TAMs, whereas pathways related to uptake and presentation of exogenous peptide antigens through MHC class 2 molecules were upregulated in M2 macrophages (Fig. 1d, e). These results suggest that although TAMs and M2 macrophages share many phenotypes, TAMs exhibit a gene expression pattern distinct from that of M2 macrophages.

### Glutamine-derived aspartate is required for TAM polarization

**Fig. 2:**
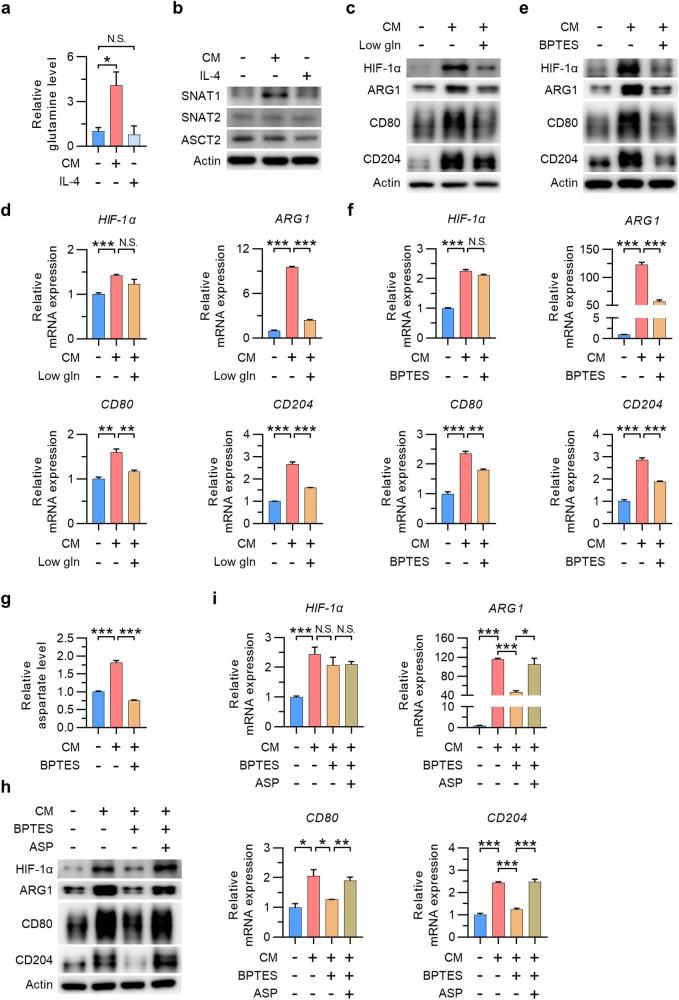
Glutamine-derived aspartate promotes TAM polarization. **a** Relative glutamine level in TAMs and M2 macrophages. **b** Levels of SNAT1, SNAT2, and ASCT2 in TAMs and M2 macrophages. **c**, **d** Protein levels (**c**) and relative expression levels of the mRNAs (**d**) encoding HIF-1α, ARG1, CD80, and CD204 in TAMs and low-glutamine-conditioned TAMs. **e**, **f** HIF-1α, ARG1, CD80, and CD204 mRNA (**e**) and protein (**f**) expression levels in TAMs in the presence or absence of BPTES. **g** Relative aspartate level in TAMs in the presence or absence of BPTES. **h**, **i** Protein levels (**h**) and relative expression levels of the mRNAs encoding (**i**) HIF-1α, ARG1, CD80, and CD204 in BPTES-treated TAMs in the presence or absence of aspartate. The data are expressed as the mean ± SEM of three independent experiments. N.S. not significant; *p < 0.05; **p < 0.01; and ***p < 0.001. CM conditioned medium, Gln glutamine, Asp aspartate.

To examine the role of glutamine metabolism in TAM polarization, we measured the levels of glutamine and glutamine transporters in TAMs and M2 macrophages. The intracellular glutamine concentration and the protein level of SNAT1, a well-known glutamine transporter, were increased in TAMs but not in M2 macrophages (Fig. 2a, b). In contrast, the levels of the glutamine transporters SNAT2 and ASCT2 remained unchanged in both TAMs and M2 macrophages. In addition, the increases in ARG1, CD80, and CD204 mRNA and protein expression in TAMs were attenuated in response to low glutamine or BPTES treatment (Fig. 2c–f). Interestingly, the upregulation of HIF-1α in TAMs decreased in response to low glutamine or BPTES treatment at the protein level only, apparently because posttranscriptional regulation of HIF-1α is dependent on glutamine availability. Next, we explored whether glutamine metabolism is required for the regulation of HIF-1α expression and TAM polarization. Glutamine is metabolized to α-ketoglutarate (α-KG) via glutamate, which can be incorporated into the TCA cycle and also serves as a precursor for the biosynthesis of glutathione (GSH), a potent antioxidant^16,33^. Notably, BPTES-mediated suppression of HIF-1α expression and TAM polarization were not reversed by supplementation with dimethyl α-ketoglutarate (DKG) or GSH (Supplementary Fig. 1a, b). However, the level of aspartate, which can be generated from glutamine via GOT1, was increased in TAMs, although this increase was reversed upon BPTES treatment (Fig. 2g). Furthermore, aspartate reversed the BPTES-induced reductions in the HIF-1α, ARG1, CD80, and CD204 levels in TAMs (Fig. 2h, i). These results indicate that glutamine-derived aspartate, rather than α-KG or GSH, is responsible for regulating HIF-1α expression and TAM polarization. Neither BPTES nor aspartate affected TAM viability or the iNOS level (Supplementary Fig. 1c, d). Given that the expression of HIF-1α mRNA was unchanged by treatment with either BPTES or aspartate, these data suggest that glutamine-derived aspartate is required for the posttranscriptional regulation of HIF-1α expression and TAM polarization.

### Glutamine-derived aspartate upregulates HIF-1α expression via the polyamine synthesis pathway and the hypusination of eIF5A

**Fig. 3:**
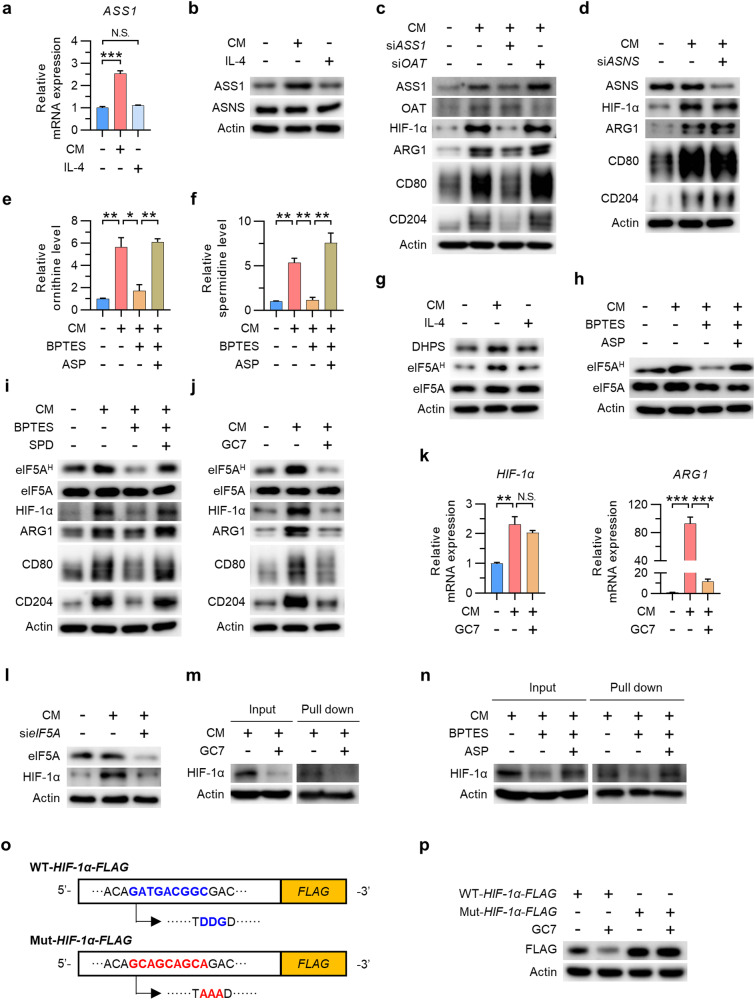
Glutamine-derived aspartate upregulates HIF-1α expression via the polyamine synthesis pathway and hypusination of eIF5A. **a** Relative mRNA expression of ASS1 in TAMs and M2 macrophages. **b** Levels of ASS1 and ASNS in TAMs and M2 macrophages. **c** Levels of HIF-1α, ARG1, CD80, and CD204 in TAMs with or without silencing of ASS1 or OAT. **d** Levels of HIF-1α, ARG1, CD80, and CD204 in TAMs with or without silencing of ASNS. **e**, **f** Relative ornithine (**e**) and spermidine (**f**) levels in TAMs in the presence or absence of BPTES or aspartate. **g** Level of eIF5A hypusinated by DHPS in TAMs and M2 macrophages. **h** Level of hypusinated eIF5A in TAMs in the presence or absence of BPTES or aspartate. **i** Levels of hypusinated eIF5A, HIF-1α, ARG1, CD80, and CD204 in TAMs in the presence or absence of BPTES or spermidine. **j** Levels of hypusinated eIF5A, HIF-1α, ARG1, CD80, and CD204 in TAMs in the presence or absence of GC7. **k** Relative expression of the mRNAs encoding HIF-1α and ARG1 in TAMs in the presence or absence of GC7. **l** Levels of eIF5A and HIF-1α in TAMs with or without silencing of eIF5A. **m**, **n** Nascent proteins expressed by TAMs in the presence or absence of GC7 (**m**), as well as in the presence or absence of BPTES or aspartate (**n**), were labeled for 4 h with L-azidohomoalanine (AHA), biotinylated via a click reaction, and pulled down for western blotting. **o** Schematic showing the WT mHIF-1α-FLAG and mutant mHIF-1α-FLAG constructs. **p** Level of FLAG in WT mHIF-1α-FLAG- or mutant mHIF-1α-FLAG-transfected HEK293T cells in the presence or absence of GC7. The data are expressed as the mean ± SEM of three independent experiments. N.S. not significant, *p < 0.05; **p < 0.01; and ***p < 0.001. CM conditioned medium, ASS1 argininosuccinate synthase, ASNS asparagine synthetase, OAT ornithine aminotransferase, ASP aspartate, SPD spermidine.

Glutamine-derived aspartate is either converted to argininosuccinate via argininosuccinate synthase 1 (ASS1) and then incorporated into the urea cycle or used by asparagine synthetase (ASNS) to synthesize asparagine^21,22,34^. To confirm the metabolic pathway through which glutamine-derived aspartate induces TAM polarization, we investigated changes in the expression of ASS1 and ASNS in TAMs. The results showed that ASS1 was upregulated in CM-treated macrophages, whereas ASNS was not (Fig. 3a, b). Moreover, siRNA-mediated silencing of ASS1 attenuated the CM-induced upregulation of HIF-1α and the TAM polarization of CM-treated macrophages (Fig. 3c; Supplementary Fig. 2a) without affecting cell viability or the iNOS level (Supplementary Fig. 2b, c); however, inhibiting ASNS did not alter the HIF-1α level or TAM polarization (Fig. 3d; Supplementary Fig. 2d). Considering the significant influence of ASS1 on the urea cycle and the polyamine pathway^35^, we hypothesized that glutamine-derived aspartate is responsible for increasing HIF-1α expression and TAM polarization via polyamine synthesis. Since glutamine catabolism contributes to the generation of ornithine via ornithine aminotransferase (OAT)-induced metabolism of pyrroline-5-carboxylate (P5C)^36^, we next checked whether silencing OAT reduces the HIF-1α level in TAMs. Knocking down OAT did not affect the HIF-1α level or TAM polarization (Fig. 3c; Supplementary Fig. 2e). We also observed elevated ornithine and spermidine levels in CM-treated macrophages, supporting the increased activity of the polyamine synthesis pathway in TAMs (Fig. 3e, f). Furthermore, the increases in the ornithine and spermidine levels in TAMs were attenuated by BPTES but were restored after supplementation with aspartate (Fig. 3e, f). Given that spermidine is a substrate for DHPS, the rate-limiting enzyme required for the hypusination of eIF5A^24^, we next investigated whether the glutamine/aspartate-derived polyamine pathway participates in eIF5A hypusination and TAM polarization. As expected, CM-treated TAMs exhibited increased levels of both DHPS and hypusinated eIF5A, whereas IL-4-induced M2 macrophages did not (Fig. 3g). CM-induced eIF5A hypusination was inhibited by BPTES but was restored after aspartate supplementation, confirming the requirement for glutamine-derived aspartate for eIF5A hypusination (Fig. 3h). We also observed that supplementation with spermidine restored TAM polarization, eIF5A hypusination and the HIF-1α protein level, all of which were attenuated by BPTES treatment (Fig. 3i). Finally, inhibition of eIF5A hypusination by GC7 reduced the HIF-1α protein level and attenuated TAM polarization without affecting cell viability or the iNOS level (Fig. 3j, k; Supplementary Fig. 2f–h), while treatment with GC7 did not alter the expression of HIF-1α mRNA in TAMs (Fig. 3k). Accordingly, we needed to confirm whether the formation of hypusinated, active eIF5A mediated by glutamine-derived aspartate contributes significantly to translational regulation of HIF-1α expression in TAMs. As expected, we observed a reduction in the HIF-1α protein level in TAMs when eIF5A was knocked down (Fig. 3l). GC7 decreased the nascent HIF-1α protein level, as assessed by measuring the abundance of newly synthesized HIF-1α proteins labeled with L-azidohomoalaine (AHA) in the presence or absence of GC7 (Fig. 3m). Moreover, aspartate supplementation restored the nascent HIF-1α protein level, which was reduced by BPTES treatment (Fig. 3n). Hypusination of eIF5A helps to overcome ribosome stalling on mRNAs encoding sequences containing polyproline motifs, e.g., DDG, DVG, and GGT^37^. Thus, we hypothesized that hypusinated eIF5A recognizes the amino acid motif DDG in HIF-1α, thereby increasing its translation efficiency. To this end, we overexpressed wild-type (DDG) and mutant (AAA) HIF-1α and then investigated the responses of these forms of HIF-1α to GC7-mediated inhibition of eIF5A hypusination. Although wild-type HIF-1α protein expression was downregulated by GC7 treatment, the expression of the mutant form was not downregulated, suggesting that the DDG motif within HIF-1α is regulated by hypusinated eIF5A (Fig. 3o, p).

### HIF-1α-dependent glycolysis promotes TAM polarization

**Fig. 4:**
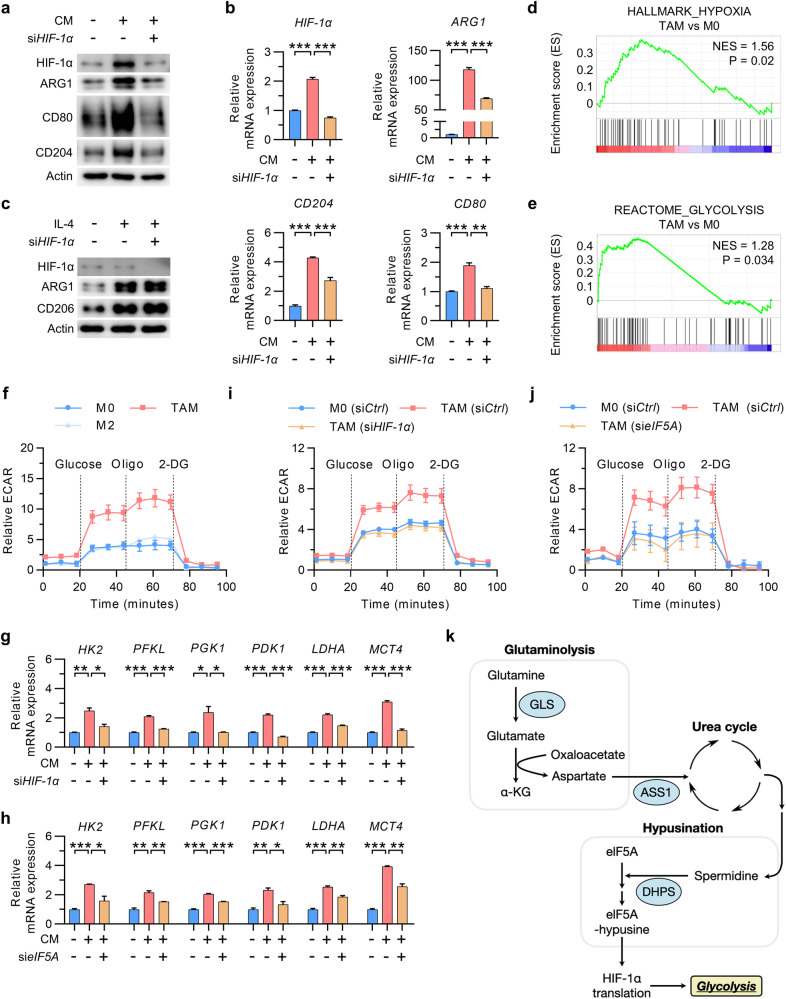
HIF-1α-dependent glycolysis is required for TAM polarization. **a**, **b** HIF-1α, ARG1, CD80, and CD204 protein (**a**) and mRNA (**b**) expression in TAMs with or without silencing of HIF-1α. **c** Levels of HIF-1α, ARG1, and CD206 in M2 macrophages with or without silencing of HIF-1α. **d**, **e** Gene set enrichment analysis of the genes related to hypoxia (**d**) and glycolysis (**e**) in TAMs versus M0 macrophages. **f** Extracellular acidification rate (ECAR) in TAMs and M2 macrophages. **g**, **h** Relative expression of the mRNAs encoding glycolysis-related HIF-1α target genes in TAMs with or without silencing of HIF-1α (**g**) or eIF5A (**h**). **i**, **j** The ECAR in TAMs with or without silencing of HIF-1α (**i**) or eIF5A TAMs (**j**). **k** Schematic showing the role of the glutamine-derived aspartate-mediated eIF5A hypusination axis in increasing glycolysis in TAMs. The data are expressed as the mean ± SEM of three independent experiments. *p < 0.05, **p < 0.01, and ***p < 0.001. CM conditioned medium, Oligo oligomycin A, 2-DG 2-deoxy-D-glucose, HK2 hexokinase 2, PFKL phosphofructokinase, liver type, PGK1 phosphoglycerate kinase 1, PDK1 pyruvate dehydrogenase kinase 1, LDHA lactate dehydrogenase A, MCT4 monocarboxylate transporter 4.

Next, to determine whether HIF-1α plays a metabolic role in TAMs that is different from its role in M2 macrophages, we examined the effects of silencing HIF-1α on the polarization of TAMs and M2 macrophages. The results showed that silencing HIF-1α suppressed the polarization of TAMs without affecting cell viability or the iNOS level (Fig. 4a, b; Supplementary Fig. 3a–c). In contrast, HIF-1α was not upregulated in IL-4-treated M2 macrophages, and silencing HIF-1α did not alter the M2 polarization of macrophages (Fig. 4c). RNA-seq profiling of macrophages revealed that the expression of genes involved in hypoxia- and glycolysis-related pathways was increased in TAMs but not in IL-4-treated macrophages (Fig. 4d, e; Supplementary Fig. 3d, e). Interestingly, TAMs exhibited an increase in glycolysis, as shown by measurement of the extracellular acidification rate (ECAR) and oxygen consumption rate (OCR); this finding suggests that TAMs exhibit the metabolic characteristics of both M1 and M2 macrophages (Fig. 4f; Supplementary Fig. 3f). In addition, the expression of enzymes required for glycolysis, i.e., HK2 and LDHA, was increased in TAMs (Fig. 4g, h). Notably, all of these changes in TAMs were reversed by silencing HIF-1α or eIF5A (Fig. 4g–j; Supplementary Fig. 3g). Moreover, we found that the presence of aspartate reversed the BPTES-induced suppression of glycolysis in CM-treated macrophages, thereby confirming the role of glutamine-derived aspartate in augmenting glycolysis in TAMs (Supplementary Fig. 3h). Additionally, silencing of either ASS1 or DHPS in TAMs suppressed glycolysis, and treatment with GC7 or silencing of DHPS in TAMs inhibited the expression of glycolytic enzyme-encoding genes (Supplementary Fig. 3i–m). Taken together, our data demonstrate that glutamine-derived aspartate in TAMs stimulates spermidine production, which increases the translation of HIF-1α via eIF5A hypusination, consequently driving an increase in glycolysis in TAMs (Fig. 4k).

### Inhibition of eIF5A hypusination in TAMs inhibits tumor growth

**Fig. 5:**
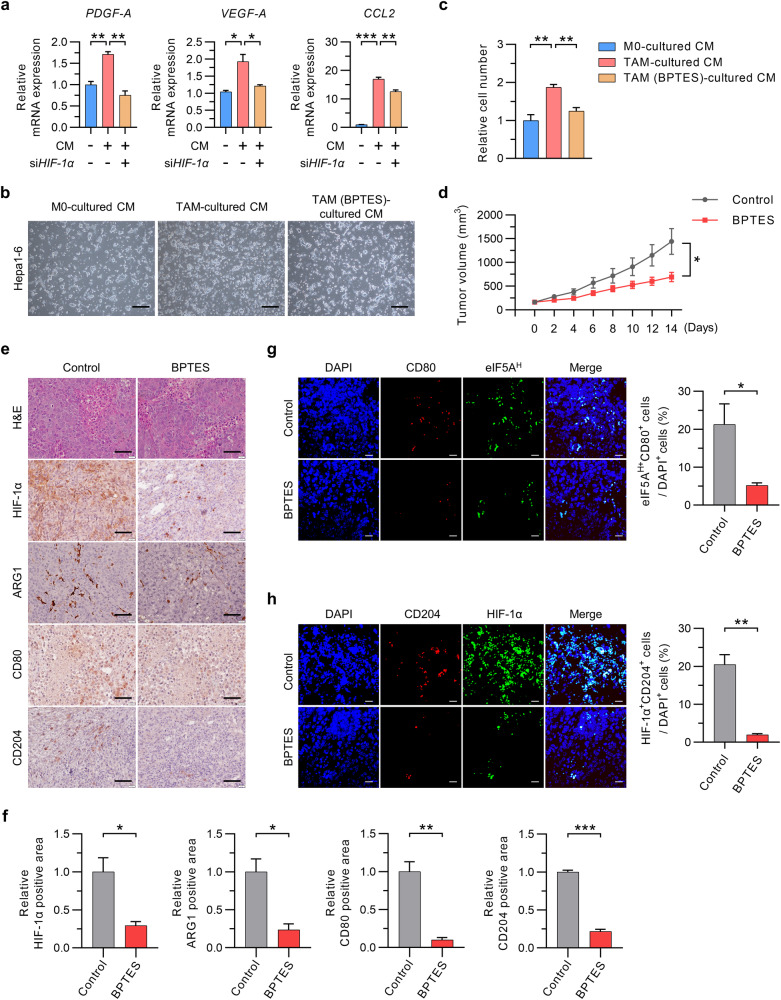
Inhibition of glutamine metabolism suppresses the growth of HCC tumors by restraining eIF5A hypusination and TAM polarization. **a** Relative expression of the mRNAs encoding PDGF-A, VEGF-A, and CCL2 in TAMs with or without silencing of HIF-1α. **b**, **c** Effects of M0 macrophages and TAMs (treated with or without BPTES) on HCC cell growth. Representative images (**b**) and relative cell numbers (**c**) are shown. **d** Growth curve of tumors in C57BL/6 mice (*n* = 12 mice per group) after BPTES treatment. **e**, **f** Hematoxylin and eosin (H&E) staining and immunohistochemical staining for HIF-1α, ARG1, CD80, and CD204 in Hepa1-6 tumor tissues from C57BL/6 mice (**e**). The numbers of immunohistochemically positive cells in tumors were determined (**f**). **g**, **h** Immunofluorescence staining of CD80 and hypusinated eIF5A (**g**) and of CD204 and HIF-1α (**h**) in Hepa1-6 tumor tissues from C57BL/6 mice treated with BPTES (left panel). Double-positive cells were quantified (right panel). Black scale bar, 60 μm; white scale bar, 20 µm. The data are expressed as the mean ± SEM of three independent experiments. *p < 0.05, **p < 0.01, and ***p < 0.001. CM conditioned medium.

**Fig. 6:**
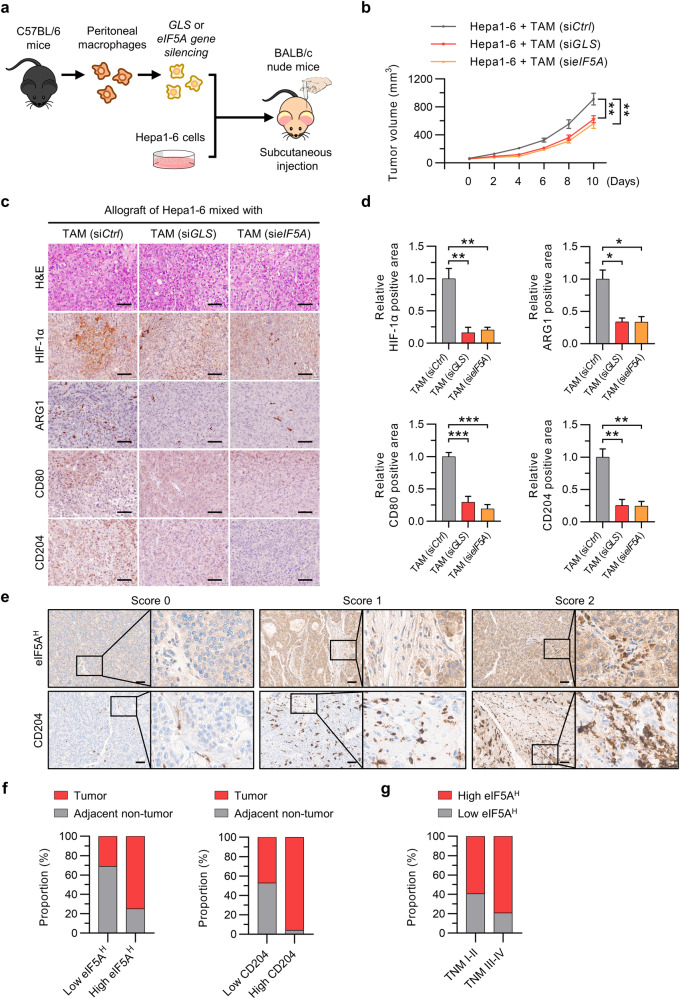
Inhibition of eIF5A hypusination suppresses HCC tumor growth, and high levels of eIF5A hypusination in tumor stromal lesions are associated with advanced stage in patients with HCC. **a** Schematic illustration of the tumor model established in BALB/c nude mice using Hepa1-6 cells mixed with GLS- or eIF5A-silenced TAMs (at a ratio of 6:1). **b** Effects of Hepa1-6 cells in combination with wild-type TAMs, GLS-knockdown TAMs, or eIF5A-knockdown TAMs in the HCC allograft model. Growth of HCC tumors in BALB/c nude mice (*n* = 10 per group). **c**, **d** Hematoxylin and eosin (H&E) staining and immunohistochemical staining for HIF-1α, ARG1, CD80, and CD204 in the tissues of allograft tumors formed from Hepa1-6 cells injected with GLS- or eIF5A-knockdown TAMs (**c**). The numbers of immunohistochemically positive cells in tumors were determined (**d**). **e** Representative images showing IHC staining for hypusinated eIF5A and CD204 in tumor stromal lesions and adjacent nontumor stromal lesions (*n* = 205) (original magnification: 400×). The degree of IHC reactivity of hypusinated eIF5A and CD204 was scored from 0 to 2+ according to the proportion of positively stained stromal cells: 0, none; 1+, <10%; 2+, >10%. **f** Distributions of tumor and adjacent nontumor tissues based on the degree of IHC reactivity of stromal hypusinated eIF5A and CD204. **g** Association between stromal hypusinated eIF5A and TNM stage. Black scale bar, 60 µm. The data are expressed as the mean ± SEM of three independent experiments. *p < 0.05, **p < 0.01, and ***p < 0.001.

The findings described above prompted further investigations to determine whether targeting glutamine metabolism or eIF5A hypusination in TAMs is a feasible approach for inhibiting HCC tumor growth. Previous studies demonstrated that growth factors and chemokines secreted by TAMs, such as PDGF-A, VEGF-A, and CCL2, increase tumor cell proliferation^7^. Here, we found that the increases in PDGF-A, VEGF-A, and CCL2 expression in CM-treated macrophages were suppressed by silencing of HIF-1α or by treatment with BPTES or GC7 (Fig. 5a; Supplementary Fig. 4a). In line with these results, CM from TAMs promoted the growth of HCC cells; however, HCC cell growth was suppressed by CM from TAMs treated with BPTES (Fig. 5b, c). Next, we tested whether targeting the glutamine-derived polyamine–hypusinated eIF5A axis in TAMs attenuates tumor growth. In the Hepa1-6 allograft model, tumor growth was significantly slower in BPTES-treated mice than in vehicle-treated mice, and there were no changes in body weight in either group (Fig. 5d; Supplementary Fig. 4b). In this model, HIF-1α expression and the number of TAMs identified according to the expression of ARG1, CD80, and CD204 were significantly lower in the tissues of BPTES-treated mice than in those of vehicle-treated mice (Fig. 5e, f). Immunofluorescence analysis of allograft tumor tissues revealed that BPTES reduced the protein levels of CD80, CD204, hypusinated eIF5A, and HIF-1α, indicating that inhibition of HCC growth can be achieved by targeting eIF5A hypusination in TAMs via regulation of glutamine metabolism (Fig. 5g, h). The antitumor efficacy of inhibiting eIF5A hypusination in TAMs was further verified in vitro and in vivo using GC7 (Supplementary Fig. 5a–g). To exclude any direct antitumor effects of BPTES or GC7, we mixed Hepa1-6 cells with GLS- or eIF5A-knockdown TAMs and then injected the mixture subcutaneously into BALB/c nude mice (Fig. 6a). The results showed that the growth of Hepa1-6 cells mixed with GLS- or eIF5A-knockdown TAMs was slower than that of Hepa1-6 cells mixed with wild-type TAMs, again with no effect on body weight (Fig. 6b; Supplementary Fig. 6a, b). We also observed that at an early time point (6 days post-injection), tumor tissues from mice implanted with Hepa1-6 cells mixed with GLS- or eIF5A-knockdown TAMs exhibited lower expression levels of HIF-1α, ARG1, CD80, and CD204 than did tumor tissues from mice implanted with Hepa1-6 cells mixed with TAMs (Fig. 6c, d). Consistent with these data, human monocyte-derived macrophages showed high protein levels of hypusinated eIF5A, HIF-1α, and CD204 upon exposure to CM from human liver cancer cells (Supplementary Fig. 6c). Finally, we measured the protein levels of hypusinated eIF5A and CD204 in stromal lesions from tumors obtained from 205 HCC patients after liver resection and compared them with those in adjacent nontumor tissues obtained from the same patients. The baseline characteristics of the 205 patients are described in Supplementary Table 1. Immunoreactivity related to eIF5A hypusination and CD204 expression in nontumor stromal lesions and tumor stromal lesions was scored from 0 to 2+ according to the intensity of staining (Fig. 6e). Immunohistochemical analysis revealed predominantly higher expression (immunoreactivity score of 2+) of eIF5A and CD204 in tumor stromal lesions than in nontumor stromal lesions; lower expression levels of these proteins were more common in noncancer stromal lesions (all *p* < 0.001) (Fig. 6f). Finally, we sought to determine whether this upregulation correlated with tumor stage in CD204-positive tumor stromal lesions. A greater percentage of patients with low levels of eIF5A hypusination in tumor stromal lesions had TNM stage I and II tumors, whereas a greater percentage of patients with high levels of eIF5A hypusination in tumor stromal lesions had TNM stage III and IV tumors (*p* = 0.012; Fig. 6g).

## Discussion

TAMs and M2 macrophages play immunosuppressive and protumorigenic roles^7,38^; therefore, several studies have investigated the roles of TAMs under M2 polarization conditions induced by IL-4^39,40^. However, recent studies have shown that TAMs have characteristics not only of M2 macrophages but also of M1 macrophages^41,42^. The present study showed that TAMs possess the metabolic characteristics of both M1 and M2 macrophages, which exhibit elevated glycolytic activity and OXPHOS activity, respectively. Notably, we demonstrated that elevated glycolysis and TAM polarization are induced by eIF5A hypusination and increased expression of HIF-α, both of which are attributed to upregulation of the glutamine-derived aspartate-mediated polyamine pathway.

TAMs are characterized by elevated glutamine utilization, which is reflected by their high expression of both glutamine transporters and catabolic enzymes^43,44^. Consistent with this, we found that the expression of glutamine transporters, as well as the intracellular glutamine level, was increased in CM-treated macrophages and that pharmacological inhibition of glutamine utilization by BPTES attenuated TAM polarization. Intriguingly, TAMs also maintained glycolytic activity, which was facilitated by the upregulation of HIF-1α, even though they utilized large amounts of glutamine. In support of our data, recent studies have demonstrated that TAMs from patients with pancreatic cancer or lung cancer exhibit increased glycolytic capacity despite competition for local glucose availability^45^. In the TME, macrophage HIF-1α expression plays a role in polarization to protumor TAMs, which promote cancer cell migration and metastasis^46^. Combining these results with our observations herein that glutamine-derived aspartate facilitates the polarization of macrophages to TAMs, accompanied by an increase in glycolytic flux, it appears that there is metabolic interplay between glucose and glutamine metabolism, which regulates TAM function via HIF-1α-dependent signaling.

Polyamines regulate protein synthesis by promoting translational elongation via eIF5A hypusination^47^. Recently, two studies reported that polyamine biosynthesis and eIF5A hypusination play different roles in M1 and M2 macrophage polarization^25,26^. One study showed that increased hypusination of eIF5A in IL-4-treated macrophages maintains TCA cycle activity, ETC integrity, and OXPHOS activity through increased translation of mitochondrial proteins, thereby maintaining M2 polarization^26^. In contrast, another study showed that hypusination of eIF5A is increased in adipose tissue macrophages via an increase in the DHPS level, which can activate NF-κB signaling and promote M1 polarization^25^. The present study showed that polyamine synthesis and eIF5A hypusination are not associated with M2 macrophages, which are considered to be protumor TAMs^38^. In a more detailed investigation, we elucidated that the expression of ASS1—the rate-limiting enzyme of arginine biosynthesis, the level of DHPS, and the level of intracellular spermidine were increased exclusively in TAMs but not in M2 macrophages. Notably, the elevation of polyamine synthesis and eIF5A hypusination in TAMs relies on the availability of glutamine-derived aspartate. Conversely, inhibiting polyamine biosynthesis and eIF5A hypusination led to loss of glycolytic activity and TAM polarization, underscoring the role of polyamine biosynthesis and eIF5A hypusination in establishing a connection between glutamine utilization and glycolysis, which is necessary for TAM polarization.

The eukaryotic translation factor eIF5A, which contains the amino acid hypusine [N epsilon-(4-amino-2-hydroxybutyl)lysine], increases the translation of proteins with non-polyproline motifs as well as those with polyproline motifs by preventing ribosomal stalling^47,48^. Accumulating evidence indicates that eIF5A mediates a range of cellular processes, including cell migration, proliferation, autophagy, and senescence^49–51^. In the context of cancer, eIF5A hypusination facilitates translation elongation of MYC and regulates the focal adhesion kinase PEAK1, both of which contribute to tumor growth^52,53^. Moreover, a recent study showed that the level of spermidine, an endogenous polyamine metabolite, impacts immune cell function by regulating eIF5A hypusination-mediated TFEB translation^50^. Given that polyamines are formed via decarboxylation of amino acids, these data suggest that targeting the metabolism of certain amino acids related to polyamine biosynthesis may attenuate protumoral cellular responses in the TME. Indeed, the present study demonstrated that the glutamine-derived aspartate-mediated eIF5A hypusination axis in TAMs affects the translation of HIF-1α mRNA via recognition of the DDG motif, a potential translation stalling motif, in HIF-1α. Based on our finding of a substantial decrease in tumor growth in vivo following a decrease in eIF5A hypusination in TAMs, targeting eIF5A could be a promising cancer treatment in the clinical setting. Especially notably, in tissues from 205 HCC patients, eIF5A hypusination in tumor stromal lesions was greater than that in adjacent nontumor stromal tissue. Moreover, the association between elevated eIF5A hypusination in the tumor stroma and advanced tumor stage suggests that targeting eIF5A hypusination in TAMs holds promise as a potent therapeutic approach.

In conclusion, the data presented herein demonstrate that glutamine-derived aspartate promotes the translation of HIF-1α through the hypusination of eIF5A, leading to TAM polarization. Thus, glutamine metabolism in TAMs supports an increase in glycolysis and maintains the capacity to promote tumor growth. Therefore, targeting eIF5A hypusination by inhibiting the glutamine-derived aspartate-mediated polyamine pathway is a promising therapeutic approach for liver cancer.

### Supplementary information

Supplementary information

WB raw data
