# Supplementary material for: Glutamine-derived aspartate is required for eIF5A hypusination-mediated translation of HIF-1α to induce the polarization of tumor-associated macrophages
Source: Exp Mol Med. 2024 May 1;56(5):1123–36. doi: 10.1038/s12276-024-01214-1 (PMC11148203; doi:10.1038/s12276-024-01214-1)
Supplement: Supplementary file 1 — Supplementary information [file 12276_2024_1214_MOESM1_ESM.pdf]

**Supplementary materials**

# Glutamine-derived aspartate is required for eIF5A hypusination-mediated translation of HIF-1α to induce the polarization of tumor-associated macrophages

Dong-Ho Kim[1]*, Yoo Na Kang[2]*, Jonghwa Jin[3], Mihyang Park[3], Daehoon Kim[1], Ghilsuk Yoon[4], Jae Won Yun[5], Jaebon Lee[6], Soo Young Park[3], Yu Rim Lee[7], Jun-Kyu Byun[8**], Yeon-Kyung Choi[7,9**], Keun-Gyu Park[1,3,9**]

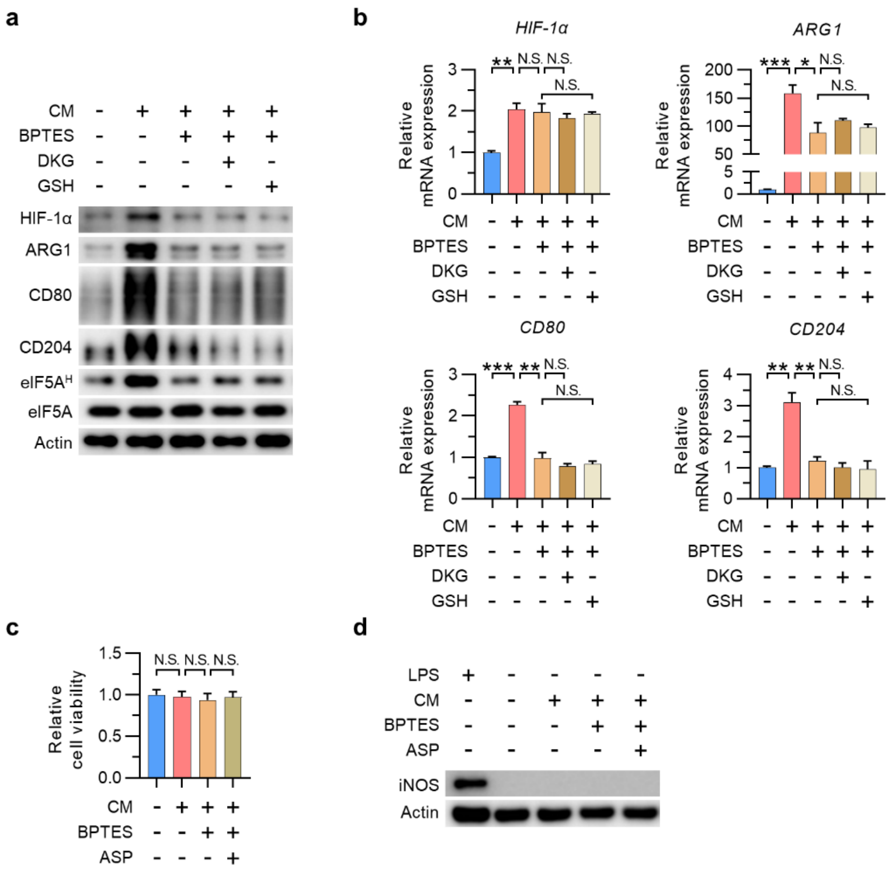


**Supplementary Fig. 1 Effects of glutamine-derived DKG or GSH on TAM polarization.**

(a, b) Protein levels (a) and relative mRNA expression (b) of HIF-1α, ARG1, CD80, CD204, and hypusinated eIF5A in TAMs in the presence or absence of BPTES, DKG, or GSH. (c) Relative viability of BPTES-treated TAMs in the presence or absence of aspartate. (d) Effects of LPS, CM, BPTES, or aspartate on the levels of iNOS in macrophages. Data are expressed as the mean ± SEM of three independent experiments. N.S., not significant, *p < 0.05, **p < 0.01, and ***p < 0.001. CM, conditioned medium; DKG, dimethyl α-ketoglutarate; Asp, aspartate; LPS, lipopolysaccharide.

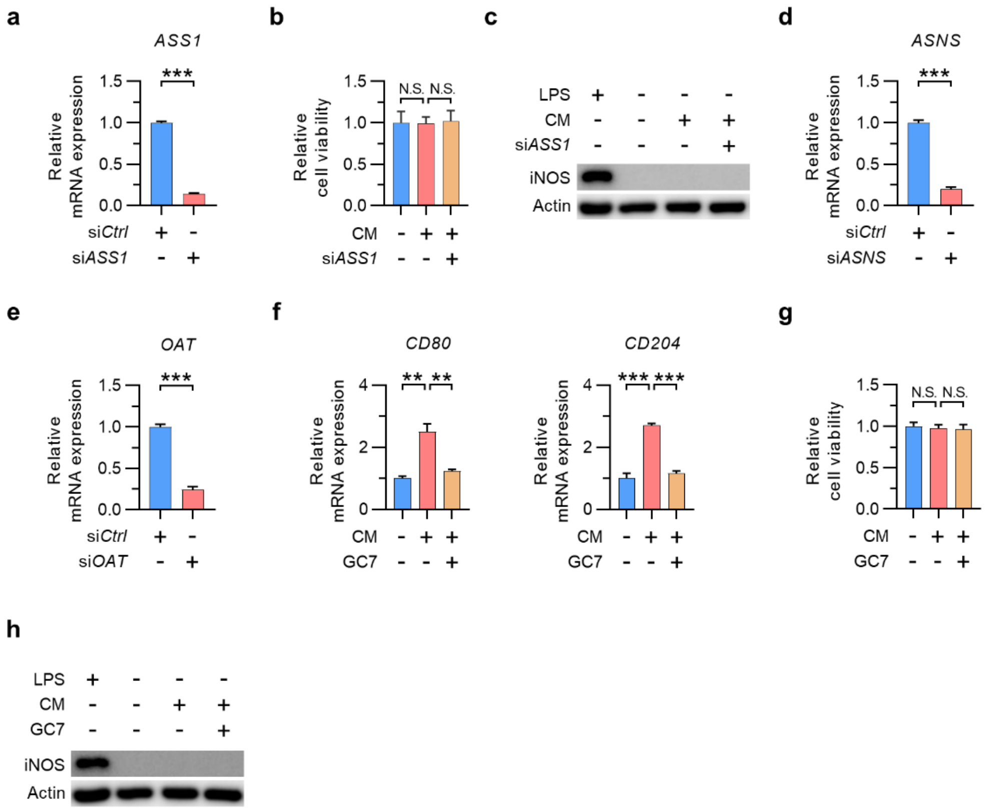


**Supplementary Fig. 2 Effects of ASS1 suppression or eIF5A hypusination inhibition on cell viability and iNOS levels in TAMs.**

(a) Validation of the ASS1 knockdown efficiency in PMs. (b) Relative viability of TAMs and ASS1-silenced TAMs. (c) Effects of LPS, CM, or silencing of ASS1 on the levels of iNOS in macrophages. (d, e) Validation of the ASNS (d) and OAT (e) knockdown efficiencies in PMs. (f) Relative expression of mRNA encoding CD80 and CD204 in TAMs in the presence or absence of GC7. (g) Relative viability of TAMS in the presence or absence of GC7. (h) Effects of LPS, CM, or GC7 on the levels of iNOS in macrophages. Data are expressed as the mean ± SEM of three independent experiments. N.S., not significant, $^{**}p < 0.01$, and $^{***}p < 0.001$. PMs, peritoneal macrophages; CM, conditioned medium; ASS1, argininosuccinate synthase; LPS, lipopolysaccharide.

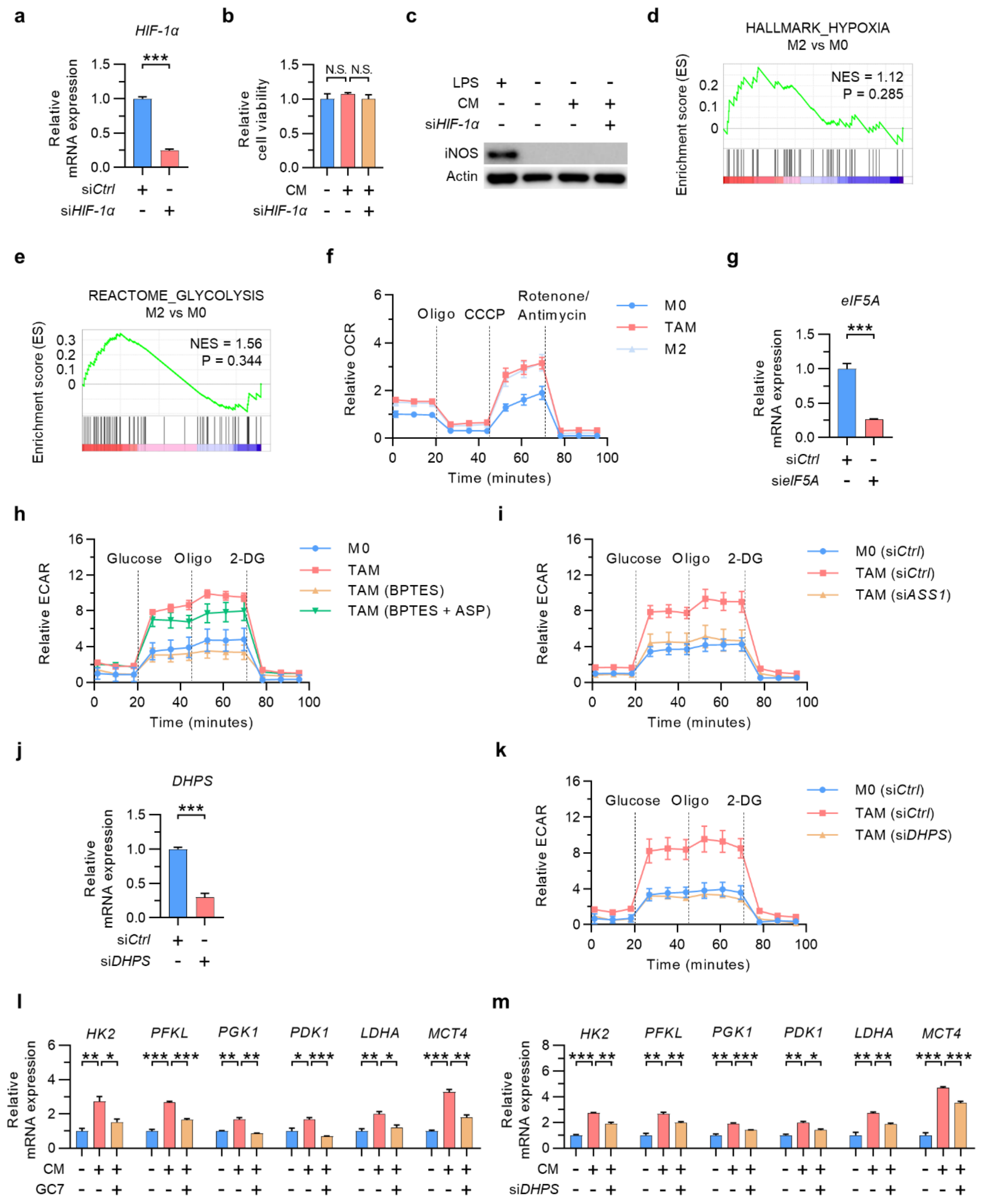


**Supplementary Fig. 3 Effects of glutamine metabolism and eIF5A hypusination inhibition on glycolysis in TAMs.**

(a) Validation of the HIF-1α knockdown efficiency in PMs. (b) Relative viability of TAMs and

HIF-1α-silenced TAMs. (c) Effects of LPS, CM, or silencing of HIF-1α on the levels of iNOS in macrophages. (d, e) Gene set enrichment analysis of genes related to hypoxia (d) and glycolysis (e) in M2 macrophages versus M0 macrophages. (f) Oxygen consumption rate (OCR) of TAMs and M2 macrophages. (g) Validation of the eIF5A knockdown efficiency in PMs. (h) Extracellular acidification rate (ECAR) level in TAMs in the presence or absence of BPTES or aspartate. (i) ECAR in TAMs and ASS1-silenced TAMs. (j) Validation of the DHPS knockdown efficiency in PMs. (k) ECAR in TAMs and DHPS-silenced TAMs. (l, m) Relative expression of mRNA encoding glycolysis-related HIF-1α target genes in TAMs, GC7-treated TAMs (l), or DHPS-silenced TAMs (m). Data are expressed as the mean ± SEM of three independent experiments. N.S., not significant. *$p < 0.05$, **$p < 0.01$, and ***$p < 0.001$. PMs, peritoneal macrophages; LPS, lipopolysaccharide; CM, conditioned medium; Oligo, Oligomycin A; CCCP, carbonyl cyanide m-chlorophenyl hydrazone; 2-DG, 2-deoxy-D-glucose; ASP, aspartate; ASS1, argininosuccinate synthase; DHPS, deoxyhypusine synthase.

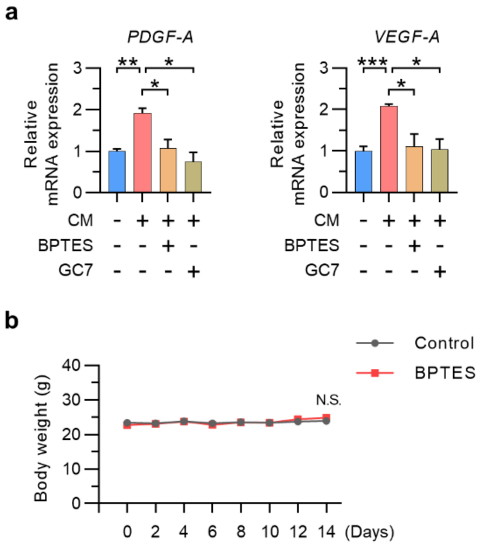


**Supplementary Fig. 4 Effects of BPTES or GC7 on production of PDGF-A, VEGF-A, and CCL2 by CM-treated macrophages.**

(a) Relative expression of mRNA encoding PDGF-A, VEGF-A, and CCL2 by TAMs in the presence or absence of BPTES or GC7. Data are expressed as the mean ± SEM of three independent experiments. (b) Body weight of C57BL/6 mice harboring Hepa1-6 cells after BPTES treatment (shown in Figure 5d). Body weight is expressed as the mean ± SEM (n=12 per group). N.S., not significant. *p < 0.05, **p < 0.01, and ***p < 0.001. PDGF-A, platelet derived growth factor subunit A; VEGF-A, vascular endothelial growth factor A; CCL2, CC motif chemokine ligand 2.

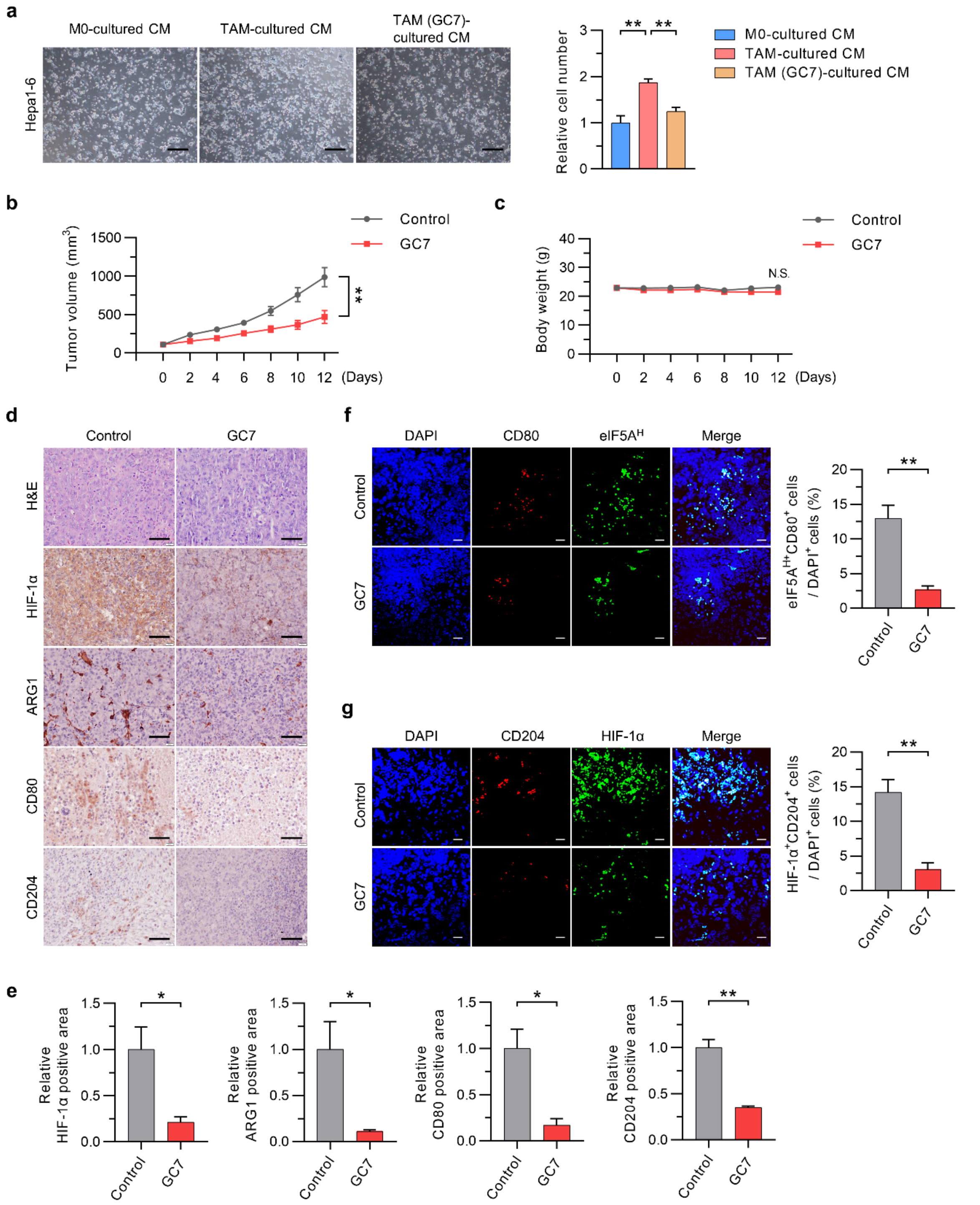


**Supplementary Fig. 5 Effects of eIF5A hypusination inhibition in TAMs on HCC tumor growth.**

(a) Effects of M0 macrophages and TAMs, treated with or without GC7, on HCC cell growth.

Representative image (left panel) and relative cell number (right panel). (b) Tumor growth curve of Hepa1-6 cells in C57BL/6 mice (n=12 per group) after GC7 treatment. (c) Body weight of C57BL/6 mice harboring Hepa1-6 cells after GC7 treatment (n=12 per group). (d, e) Hematoxylin and eosin (H&E) staining and immunohistochemical staining of HIF-1α, ARG1, CD80, and CD204 in Hepa1-6 tumor tissues from C57BL/6 mice after GC7 treatment (d). The number of immunohistochemically positive cells in tumors was counted (e). (f, g) Immunofluorescence staining of CD80 and hypusinated eIF5A (f) or CD204 and HIF-1α (g) in Hepa1-6 tumor tissues from C57BL/6 mice (left panel) after GC7 treatment. The number of double positive cells was quantified (right panel). Black scale bar, 60 μm; white scale bar, 20 μm. Data are expressed as the mean ± SEM of three independent experiments. N.S., not significant. $*p < 0.05$ and $**p < 0.01$.

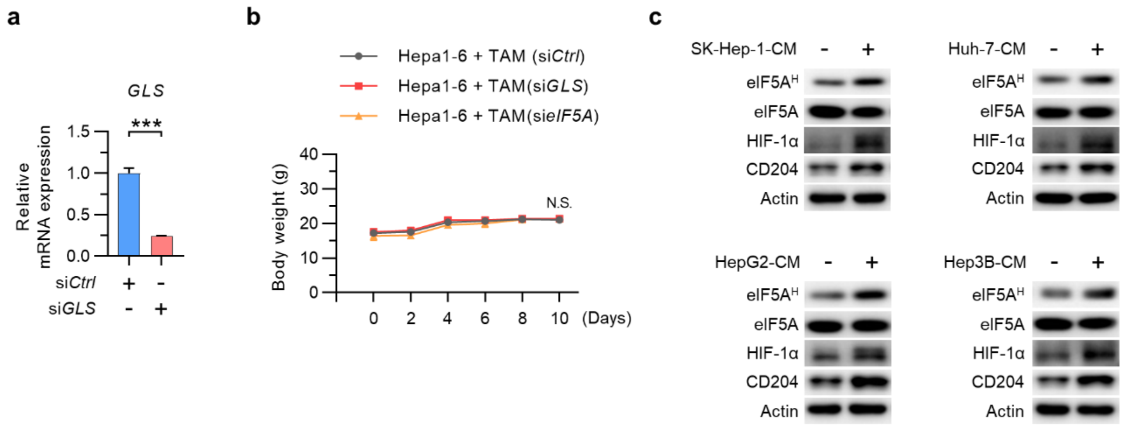


**Supplementary Fig. 6 Effects of Hepa1–6 plus TAMs, GLS-knockdown TAMs, or eIF5A-knockdown TAMs on body weight of HCC allograft mice.**

(a) Validation of the GLS knockdown efficiency in PMs. (b) Body weight of BALB/c nude mice harboring Hepa1-6 tumors treated with TAMs, GLS-knockdown TAMs, or eIF5A-knockdown TAMs. Data are expressed as the mean ± SEM (n=10 per group). (c) Levels of hypusinated eIF5A, HIF-1α, and CD204 proteins in human monocyte-derived macrophages by exposure to CM from liver cancer cells. N.S., not significant. ***p < 0.001. PMs, peritoneal macrophages; CM, conditioned medium.

**Supplementary Table. 1** Clinical characteristics of patients with resected HCC

| **Variables** | **No. of Patients** |
|---|---|
| **All cases** | 205 |
| **Gender (male/female)** | 159/46 |
| **Mean age** | 57.39 ± 10.89 |
| **HCC cause** | |
| **HBV** | 154 |
| **HCV** | 17 |
| **Alcohol** | 14 |
| **HBV + Alcohol** | 6 |
| **Other causes** | 14 |
| **Tumor Size** | |
| **< 5** | 139 |
| **≥5** | 66 |
| **TNM stage** | |
| **I-II** | 147 |
| **III-IV** | 58 |

**Supplementary Table. 2** List of antibodies used for western blot analysis and immunohistochemistry

| Name | Supplier | Catalog Number |
|---|---|---|
| Anti-Arg1 | Cell Signaling Technology, Danvers, MA, USA | Cat# 93668 |
| Anti-ASNS | Cell Signaling Technology, Danvers, MA, USA | Cat# 92479 |
| Anti-HIF-1α | Cell Signaling Technology, Danvers, MA, USA | Cat# 14179 |
| Anti-CD206 | Cell Signaling Technology, Danvers, MA, USA | Cat# 24595 |
| Anti-Phospho-Stat6 (Tyr641) | Cell Signaling Technology, Danvers, MA, USA | Cat# 56554 |
| Anti-Stat6 | Cell Signaling Technology, Danvers, MA, USA | Cat# 5397 |
| Anti-ASCT2 | Cell Signaling Technology, Danvers, MA, USA | Cat# 5345 |
| Anti-SNAT1 | Cell Signaling Technology, Danvers, MA, USA | Cat# 36057 |
| Anti-CD80 | Cell Signaling Technology, Danvers, MA, USA | Cat# 54521 |
| Anti-OAT | Novus Biologicals, Centennial, CO, USA | Cat# NBP1-83239 |
| Anti-HIF-1α | Novus Biologicals, Centennial, CO, USA | Cat# NB100-105 |
| Anti-HIF-1α | Novus Biologicals, Centennial, CO, USA | Cat# NB100-479 |
| Anti-CD204 | Novus Biologicals, Centennial, CO, USA | Cat# NBP1-00092 |
| Anti-CD204 | Abcam, Cambridge, UK | Cat# ab151707 |
| Anti-CD204 | Abcam, Cambridge, UK | Cat# ab271070 |
| Anti-eIF5A | Abcam, Cambridge, UK | Cat# ab137561 |
| Anti-iNOS | BD Bioscience, San Jose, CA, USA | Cat# 610328 |
| Anti-PD-L1 | R&D Systems, Minneapolis, MN, USA | Cat# AF1019 |
| Anti-CD80 | R&D Systems, Minneapolis, MN, USA | Cat# MAB740 |
| Anti-CD80 | Santa Cruz Biotechnology, Dallas, TX, USA | Cat# sc-376012 |
| Anti-ASS1 | Santa Cruz Biotechnology, Dallas, TX, USA | Cat# sc-365475 |
| Anti-SNAT2 | Santa Cruz Biotechnology, Dallas, TX, USA | Cat# sc-514037 |
| Anti-DHPS | ABclonal, Wuhan, China | Cat# A6367 |
| Anti-Hypusine | Sigma, St. Louis, MO, USA | Cat# ABS1064 |
| Anti-FLAG® M2 | Sigma, St. Louis, MO, USA | Cat# F1804 |
| Anti-β-Actin | Sigma, St. Louis, MO, USA | Cat# A5441 |
| Goat Anti-Rabbit IgG antibody (HRP) | GeneTex, Irvine, CA, USA | Cat# GTX213110-01 |
| Goat Anti-Mouse IgG antibody (HRP) | GeneTex, Irvine, CA, USA | Cat# GTX213111-01 |
| Donkey Anti-Goat IgG antibody (HRP) | GeneTex, Irvine, CA, USA | Cat# GTX232040-01 |
| SignalStain® Boost IHC Detection Reagent (HRP, Mouse) | Cell Signaling Technology, Danvers, MA, USA | Cat# 8125 |
| SignalStain® Boost IHC Detection Reagent (HRP, Rabbit) | Cell Signaling Technology, Danvers, MA, USA | Cat# 8114 |

| | | |
|---|---|---|
| SignalStain® Boost IHC Detection Reagent (HRP, Rat) | Cell Signaling Technology, Danvers, MA, USA | Cat# 72838 |
| Alexa Fluor 568, Goat anti-Rabbit IgG | Thermo Fisher Scientific, Waltham, MA, USA | Cat# A-11036 |
| Alexa Fluor 488, Goat anti-mouse IgG | Thermo Fisher Scientific, Waltham, MA, USA | Cat# A-11001 |

**Supplementary Table. 3** List of primers used for PCR

| Gene | Primer | Primer sequence |
|---|---|---|
| Mouse Arg1 | Forward | CCACAGTCTGGCAGTTGGAA |
| | Reverse | TGTGAGCATCCACCCAAATG |
| Mouse Arg2 | Forward | ACGGGCAAATTCCTTGCGT |
| | Reverse | TGGACCATATTCCACTCCTAGC |
| Mouse ASS1 | Forward | GCCTTGCATAGCTCGCAGA |
| | Reverse | GGACCTGGTCATTCCCCTTT |
| Mouse ASNS | Forward | TCAAGGAGGCCCAAGTTCAG |
| | Reverse | GGCTGTCCTCCATGCCAATA |
| Mouse CCL2 | Forward | TAAAAACCTGGATCGGAACCAAA |
| | Reverse | GCATTAGCTTCAGATTTACGGGT |
| Mouse CCL17 | Forward | TACCATGAGGTCACTTCAGATGC |
| | Reverse | GCACTCTCGGCCTACATTGG |
| Mouse CCL24 | Forward | GGCAATAGCACCGAGGTTTA |
| | Reverse | TTCTCAGAGCGGATGAAGGT |
| Mouse CD80 | Forward | TGCTGCTGATTCGTCTTTCAC |
| | Reverse | GAGGAGAGTTGTAACGGCAAG |
| Mouse CD204 | Forward | TGGAGGAGAGAATCGAAAGCA |
| | Reverse | CTGGACTGACGAAATCAAGGAA |
| Mouse CD206 | Forward | GAGGGAAGCGAGAGATTATGGA |
| | Reverse | GCCTGATGCCAGGTTAAAGCA |
| Mouse DHPS | Forward | AGGAGATCAACAACCCAGACT |
| | Reverse | AGATCATGTCACCCAGTGAGC |
| Mouse eIF5A | Forward | TCCCCAACATCAAACGGAAT |
| | Reverse | CCTCTCGTACCTCCCCACTGT |
| Mouse GLS | Forward | CTACAGGATTGCGAACATCTGAT |
| | Reverse | ACACCATCTGACGTTGTCTGA |
| Mouse HIF-1α | Forward | ACTGCACGGGCCATATTCAT |
| | Reverse | GCACGTCATGGGTGGTTTCT |
| Mouse IL-1β | Forward | GAGCACCTTCTTTTCCTTCATCTT |
| | Reverse | TCACACACCAGCAGGTTATCATC |
| Mouse MGL2 | Forward | TTCAAGAATTGGAGGCCACT |
| | Reverse | CAGACATCGTCATTCCAACG |
| Mouse OAT | Forward | CCACAGATCCGACCAGTTATGA |
| | Reverse | TGCGGGCAGATCGTTATATG |
| Mouse PD-L1 | Forward | GCCTCAGCACAGCAACTTCA |
| | Reverse | GGCAGCATTTCCCTTCAAAA |
| Mouse VEGF-A | Forward | CAGGCTGCTGTAACGATGAA |
| | Reverse | GCATTCACATCTGCTGTGCT |
| Mouse PDGF-A | Forward | TGTGCCCATTCGCAGGAAG |
| | Reverse | GAGGTATCTCGTAAATGACCGTC |
| Mouse HK2 | Forward | ACGGAGCTCAACCAAAACCA |
| | Reverse | AACCGCCTAGAAATCTCCAGAA |
| Mouse PFKL | Forward | CGGAACTATGGGACCAAACTG |
| | Reverse | CGCCCTTTACGGTAGACATCA |

| | | |
|---|---|---|
| Mouse PGK1 | Forward | AGGCTGTGGGTCGAGCTAAG |
| | Reverse | GCAAAGGCTTCCCATTCAAA |
| Mouse PDK1 | Forward | TATCCCCCGATTCAGGTTCA |
| | Reverse | CTCCCCGGTCACTCATCTTC |
| Mouse LDHA | Forward | TGAAGGACTTGGCGGATGAG |
| | Reverse | TCCATCATCTCGCCCTTGA |
| Mouse MCT4 | Forward | CATGAGTTTGGGATTGGCTACA |
| | Reverse | AGCGGTCCTGTGCCATAGAG |
| Mouse 36B4 | Forward | ACCTCCTTCTTCCAGGCTTT |
| | Reverse | CTCCAGTCTTTATCAGCTGC |

**Supplementary Table. 4** List of siRNAs and their sequences employed in this study

| Target name | Supplier | Catalog number | Duplex sequence (5' -> 3') | |
|---|---|---|---|---|
| mouse ASS1 | Bioneer, Daejeon, Korea | #11898-1 | Sense | CAG CAU UAA UUG UUG UGA UdTdT |
| | | | Antisense | AUC ACA ACA AUU AAU GCU GdTdT |
| mouse GLS | Bioneer, Daejeon, Korea | #14660-1 | Sense | CAC GAU CUU GUU UCU CUG UdTdT |
| | | | Antisense | ACA GAG AAA CAA GAU CGU GdTdT |
| mouse eIF5A | Bioneer, Daejeon, Korea | #276770-1 | Sense | UGU AAG AUC GUC GAG AUG UdTdT |
| | | | Antisense | ACA UCU CGA CGA UCU UAC AdTdT |
| mouse DHPS | Bioneer, Daejeon, Korea | #330817-1 | Sense | CAG CAA GUC AAC GCC AUG AdTdT |
| | | | Antisense | UCA UGG CGU UGA CUU GCU GdTdT |
| mouse HIF-1α | Bioneer, Daejeon, Korea | #15251-1 | Sense | GUG GUU GGG UCU AAC ACU AdTdT |
| | | | Antisense | UAG UGU UAG ACC CAA CCA CdTdT |
| mouse OAT | Bioneer, Daejeon, Korea | #18242-1 | Sense | CAC GAU GCU UUC UAA ACU AdTdT |
| | | | Antisense | UAG UUU AGA AAG CAU CGU GdTdT |
| mouse ASNS | Bioneer, Daejeon, Korea | #27053-2 | Sense | GGU GGC AAA UUA UAU UGG AdTdT |
| | | | Antisense | UCC AAU AUA AUU UGC CAC CdTdT |
| Negative Control siRNA | Bioneer, Daejeon, Korea | #SN-1003 | Sequence not provided | |
