# Supplementary material for: Glutamine-derived aspartate is required for eIF5A hypusination-mediated translation of HIF-1α to induce the polarization of tumor-associated macrophages
Source: Exp Mol Med. 2024 May 1;56(5):1123–36. doi: 10.1038/s12276-024-01214-1 (PMC11148203; doi:10.1038/s12276-024-01214-1)
Supplement: Supplementary file 2 — WB raw data [file 12276_2024_1214_MOESM2_ESM.pdf]

Figure 1C

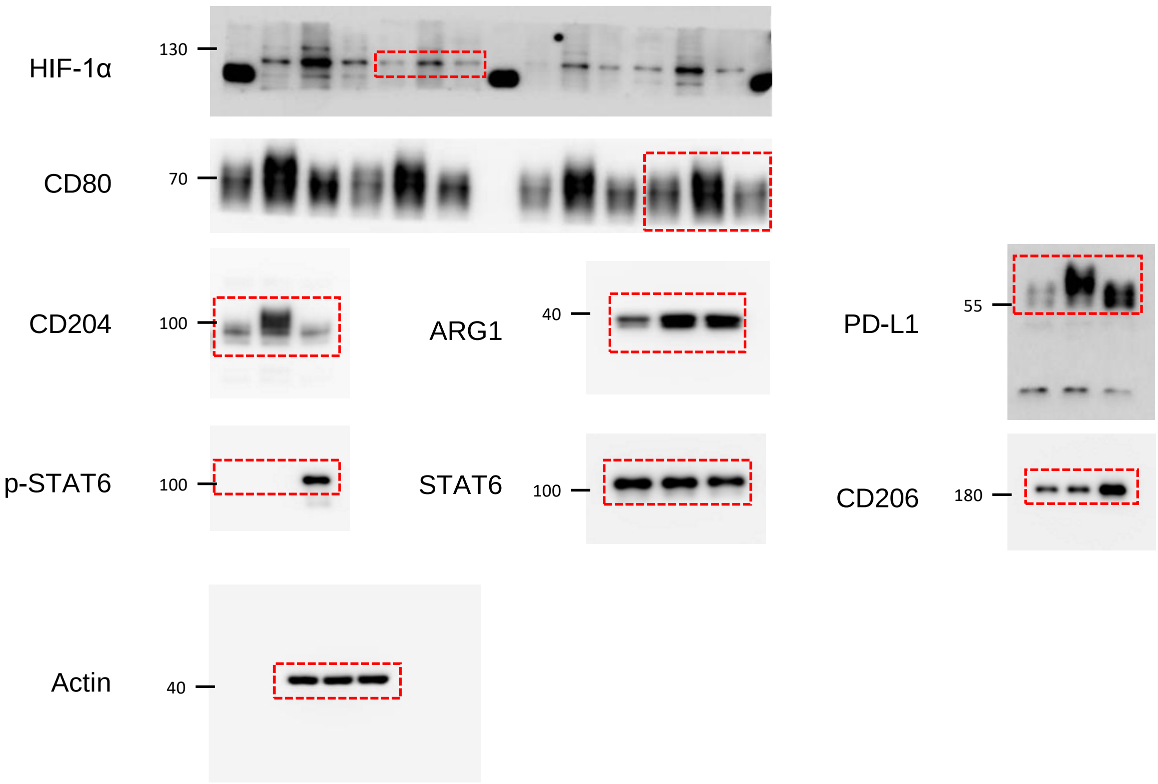
HIF-1α
130
CD80
70
CD204
100
ARG1
40
PD-L1
55
p-STAT6
100
STAT6
100
CD206
180
Actin
40

Figure 2B

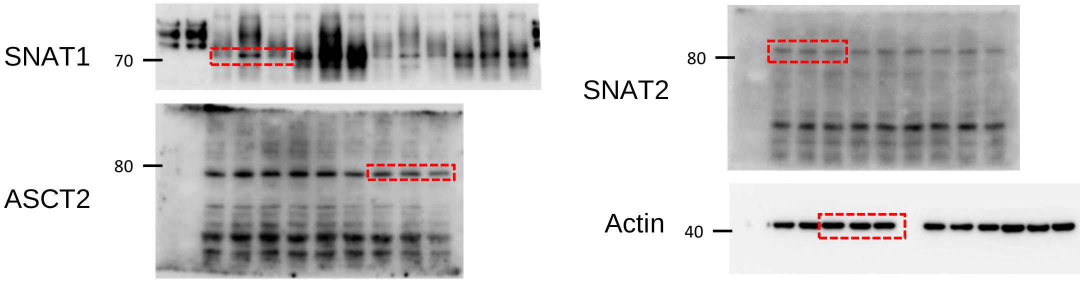

Figure 2C

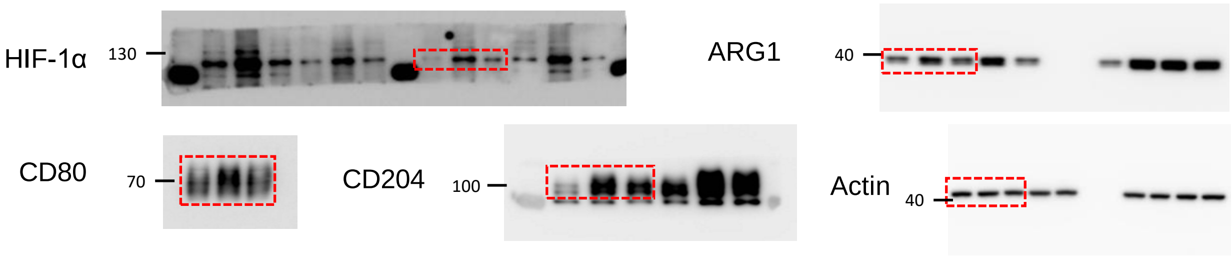

Figure 2E

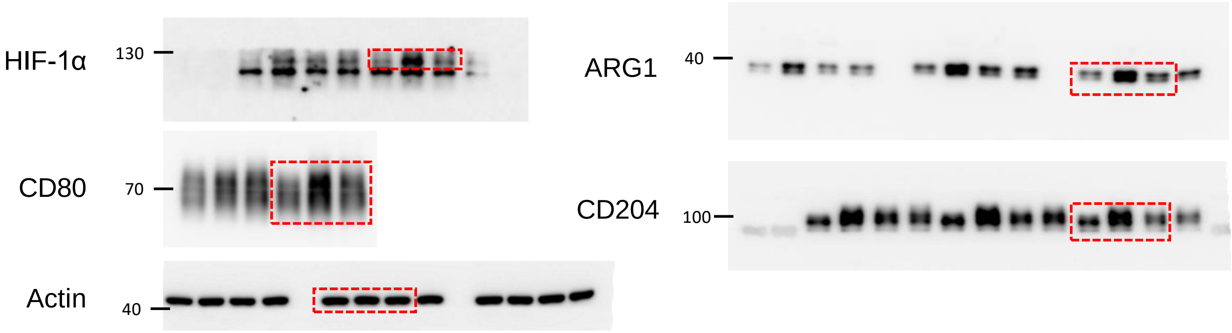

Figure 2H

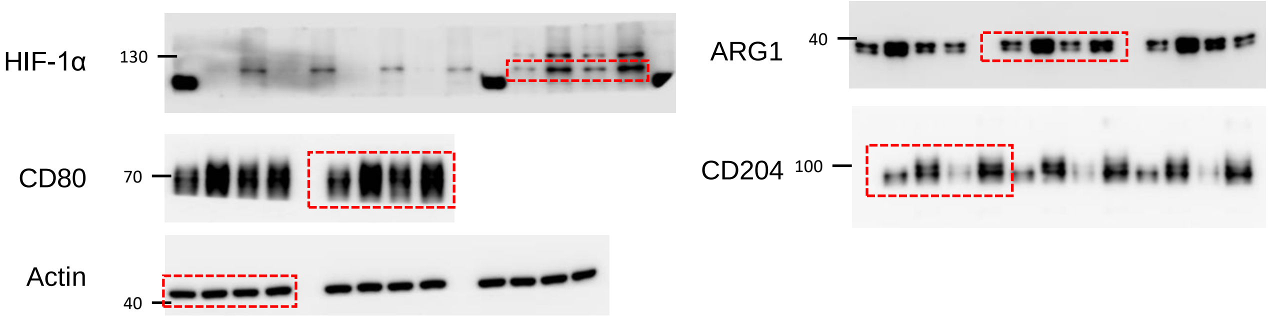

Figure 3B

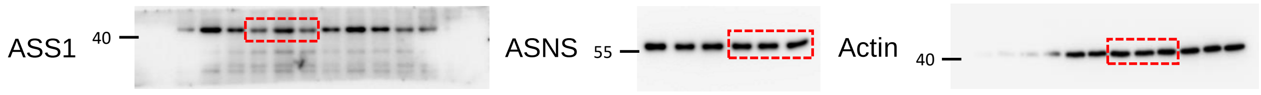

Figure 3C

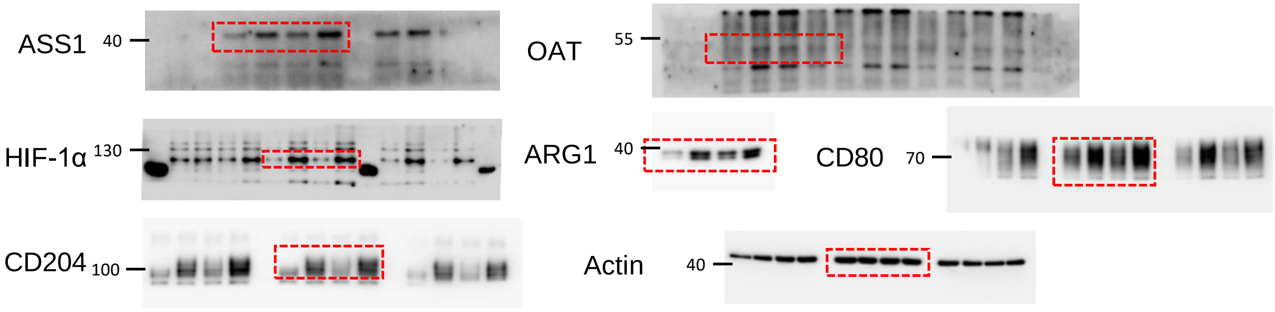

Figure 3D

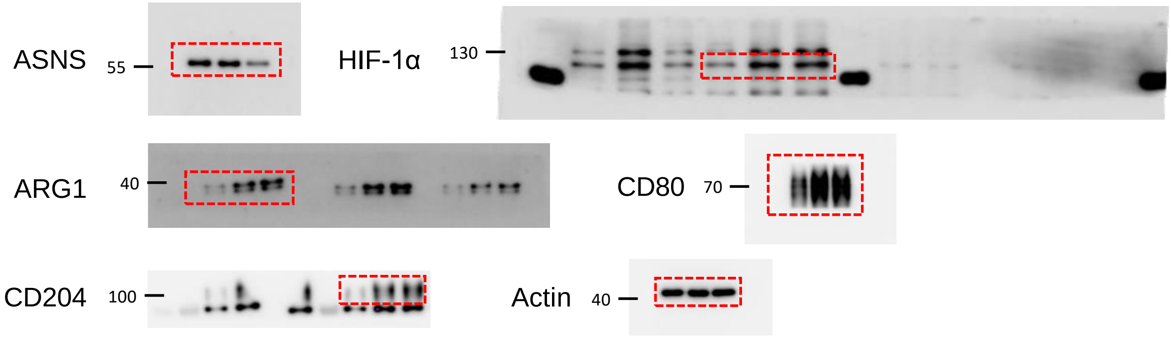

Figure 3G

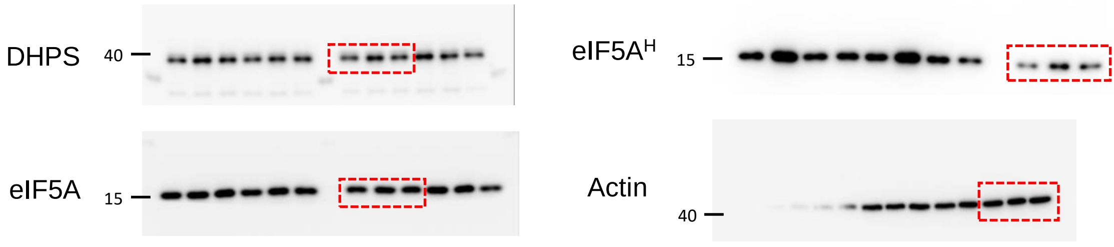

Figure 3H

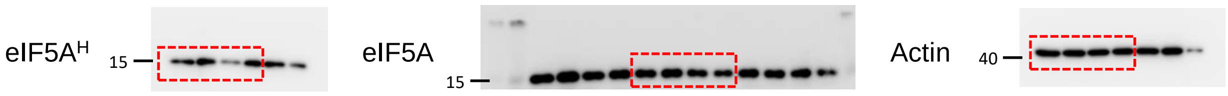

Figure 3I

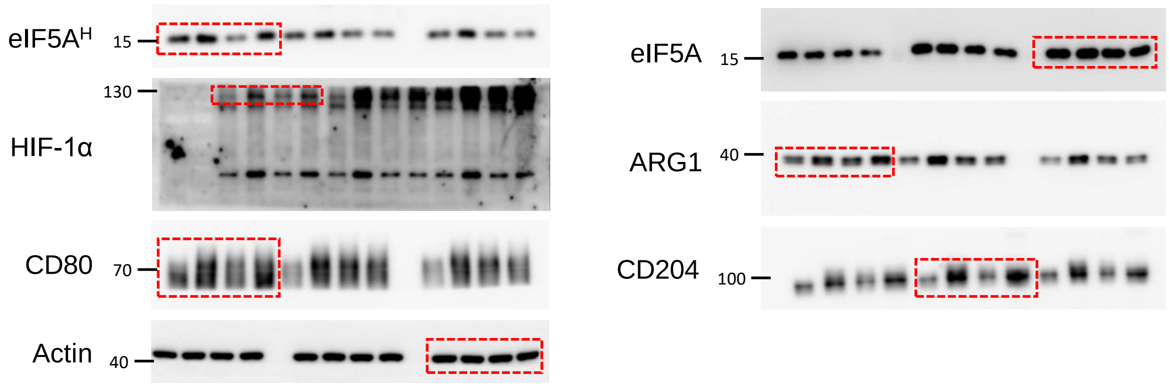

Figure 3J

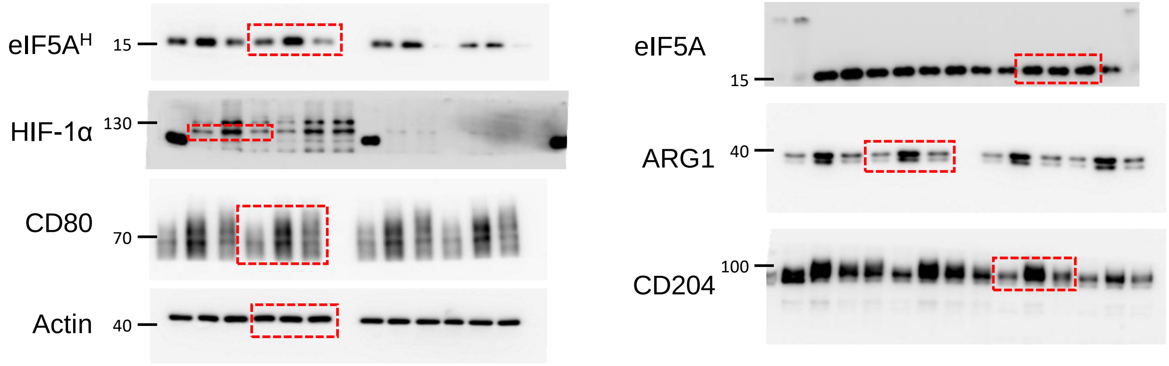

Figure 3L

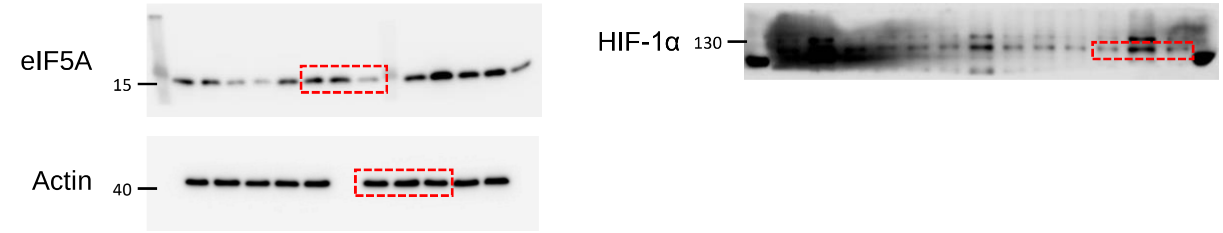

Figure 3M

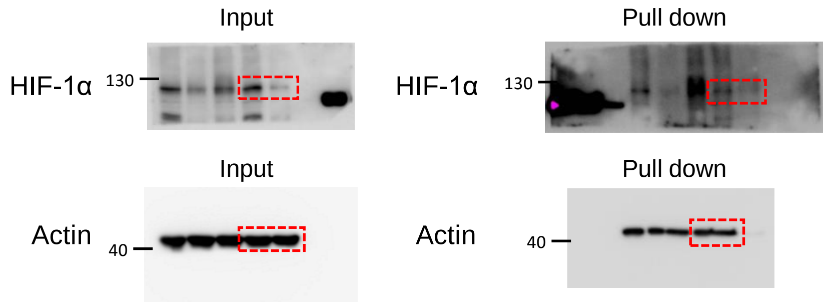

Figure 3N

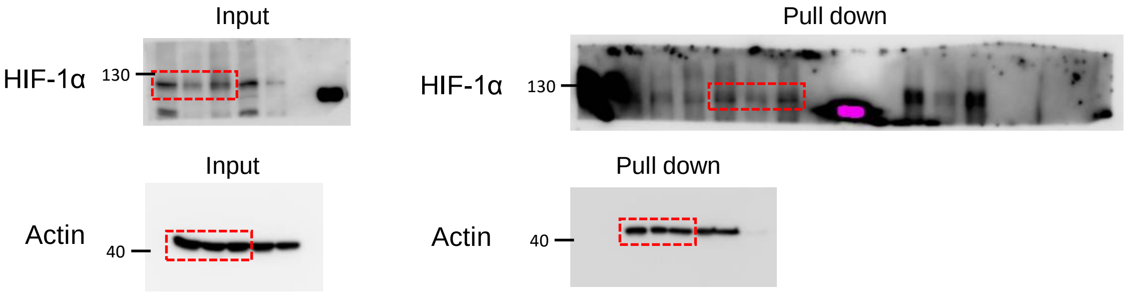

Figure 3P

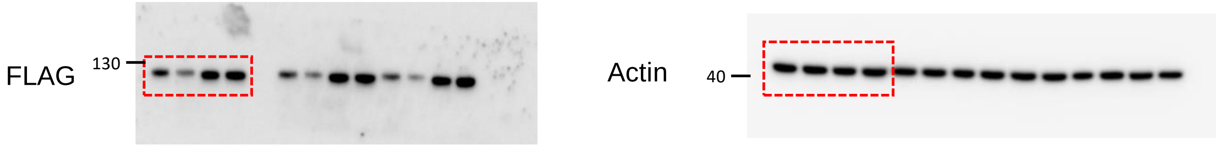

Figure 4A

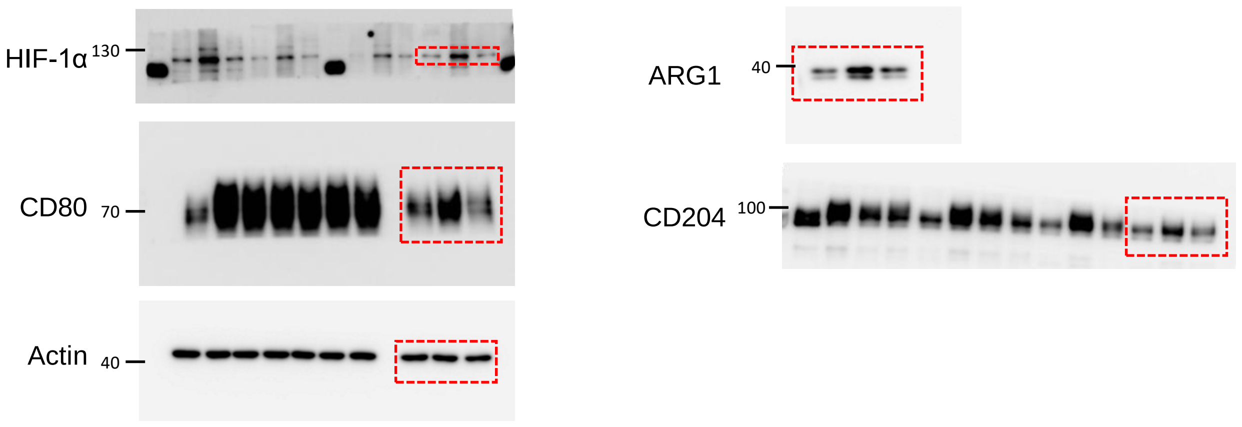

Figure 4C

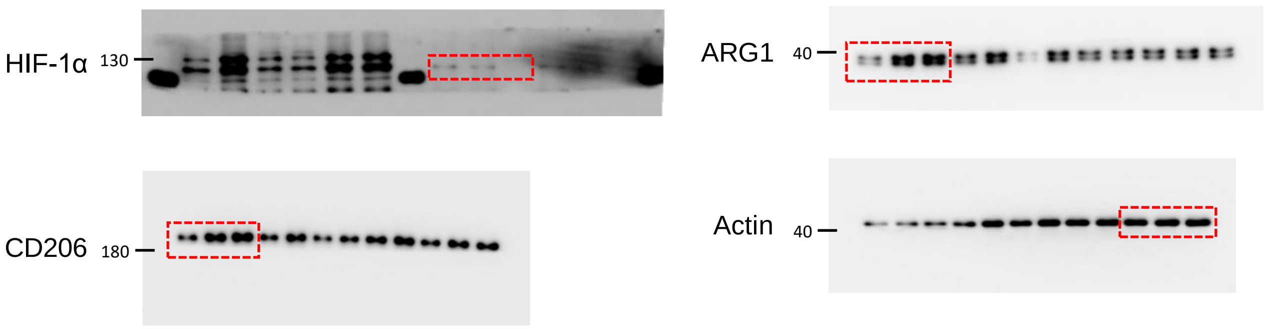

Supplementary Figure 1A

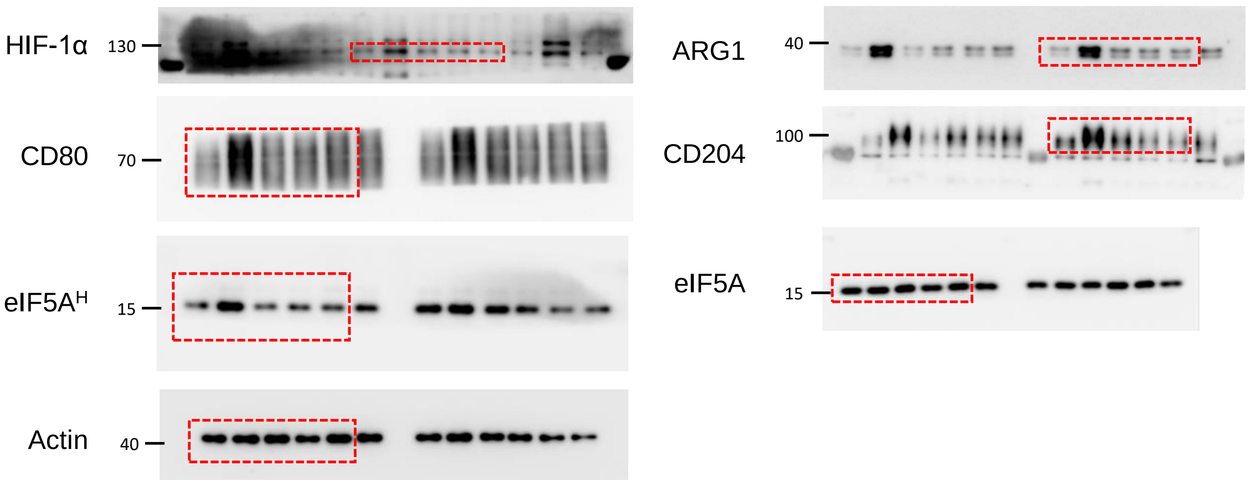

Supplementary Figure 1D

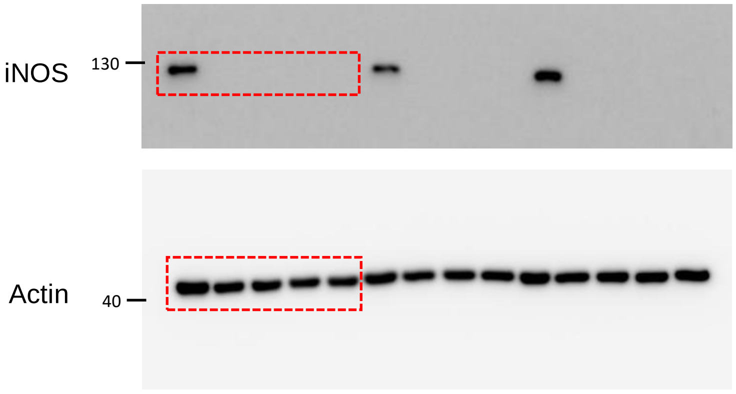

Supplementary Figure 2C

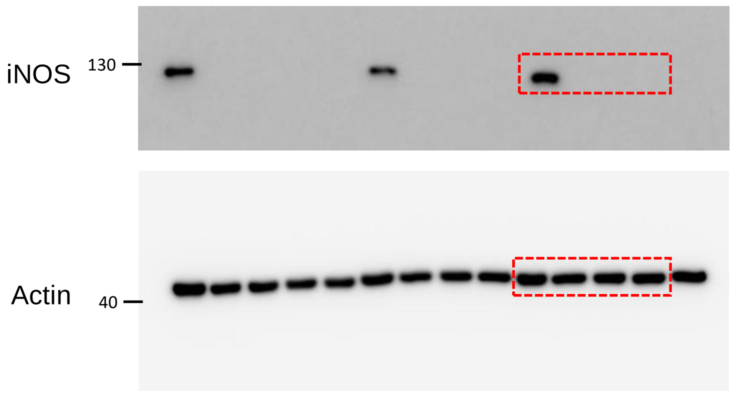

Supplementary Figure 2H

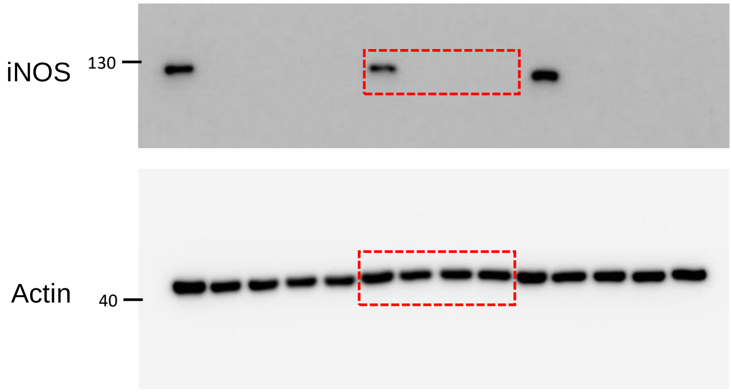

Supplementary Figure 3C

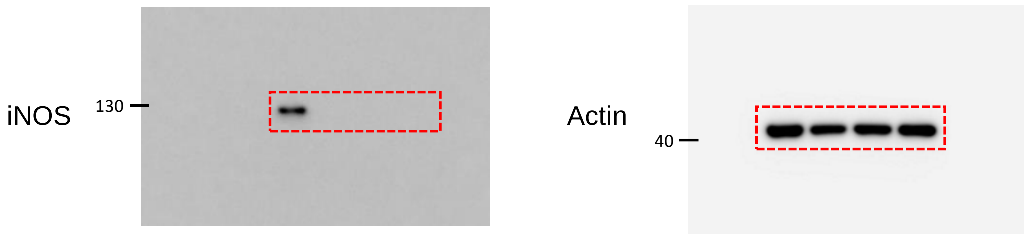

Supplementary Figure 6C

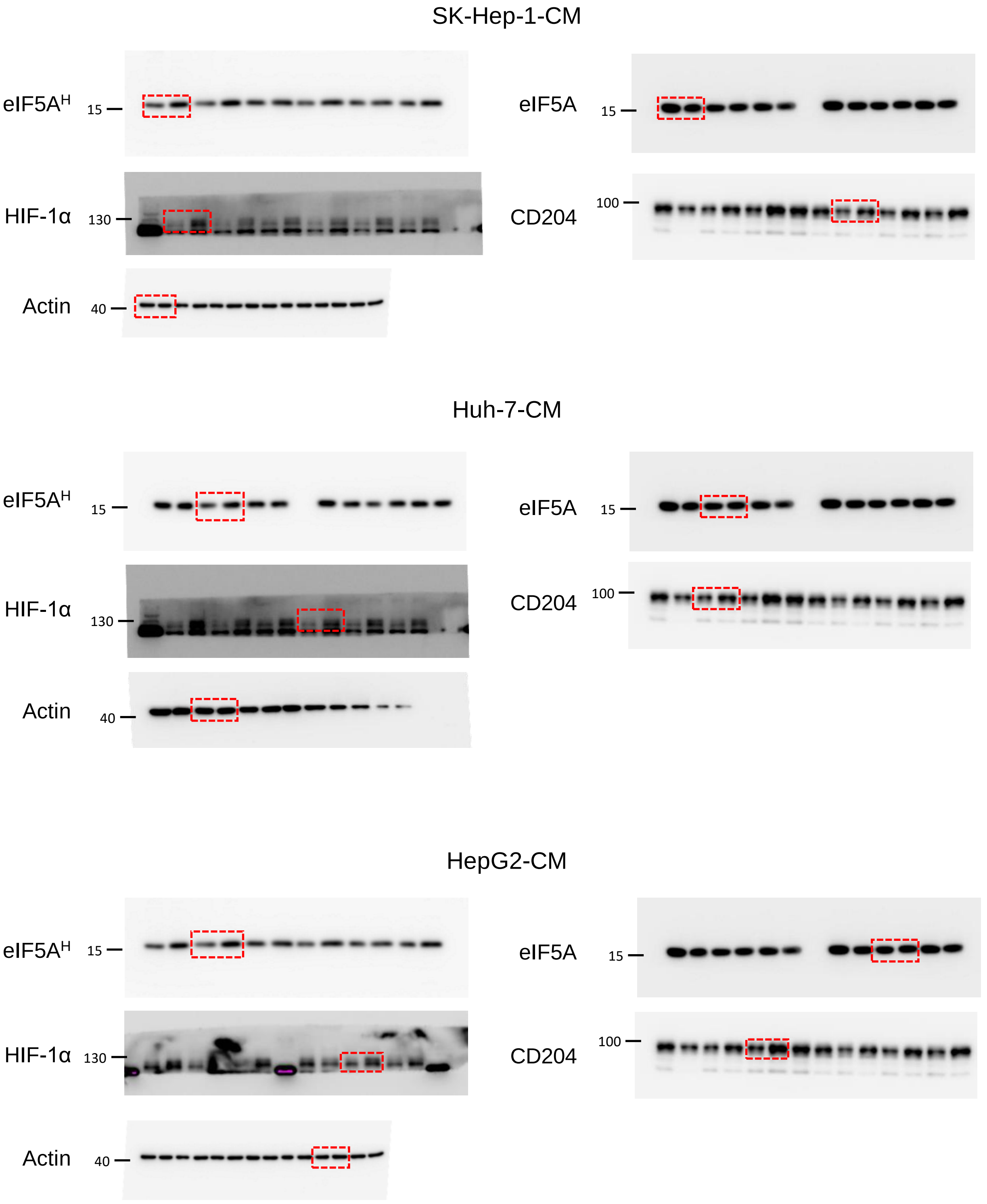

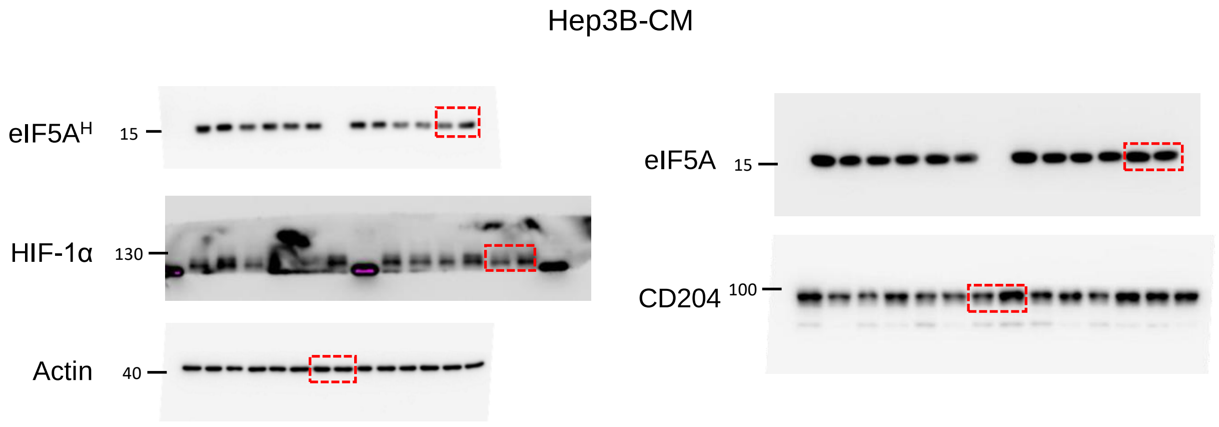
Hep3B-CM
eIF5A^H
15
HIF-1α
130
Actin
40
eIF5A
15
CD204
100
